# Quantitative relationship between cerebrovascular network and neuronal cell types in mice

**DOI:** 10.1016/j.celrep.2022.110978

**Published:** 2022-06-21

**Authors:** Yuan-ting Wu, Hannah C. Bennett, Uree Chon, Daniel J. Vanselow, Qingguang Zhang, Rodrigo Muñoz-Castañeda, Keith C. Cheng, Pavel Osten, Patrick J. Drew, Yongsoo Kim

**Affiliations:** 1 Department of Neural and Behavioral Sciences, The Pennsylvania State University, Hershey, PA 17033, USA; 2 Department of Pathology, The Pennsylvania State University, Hershey, PA 17033, USA; 3 Center for Neural Engineering, Department of Engineering Science and Mechanics, The Pennsylvania State University, University Park, PA 16802, USA; 4 Cold Spring Harbor Laboratory, Cold Spring Harbor, NY 11724, USA; 5 Department of Biomedical Engineering, The Pennsylvania State University, University Park, PA 16802, USA; 6 Department of Neurosurgery, The Pennsylvania State University, University Park, PA 16802, USA; 7 Senior author; 8 These authors contributed equally; 9 Lead contact

## Abstract

The cerebrovasculature and its mural cells must meet brain regional energy demands, but how their spatial relationship with different neuronal cell types varies across the brain remains largely unknown. Here we apply brain-wide mapping methods to comprehensively define the quantitative relationships between the cerebrovasculature, capillary pericytes, and glutamatergic and GABAergic neurons, including neuronal nitric oxide synthase-positive (nNOS^+^) neurons and their subtypes in adult mice. Our results show high densities of vasculature with high fluid conductance and capillary pericytes in primary motor sensory cortices compared with association cortices that show significant positive and negative correlations with energy-demanding parvalbumin^+^ and vasomotor nNOS^+^ neurons, respectively. Thalamo-striatal areas that are connected to primary motor sensory cortices also show high densities of vasculature and pericytes, suggesting dense energy support for motor sensory processing areas. Our cellular-resolution resource offers opportunities to examine spatial relationships between the cerebrovascular network and neuronal cell composition in largely understudied subcortical areas.

## In brief

Wu et al. generate cerebrovascular, pericyte, and neuronal cell type maps with their distribution and spatial relationship to understand the organization of brain energy infrastructure in mice. Dense cerebrovascular networks support the high energy demand of motor sensory circuits with enriched parvalbumin neurons compared with association areas with a high density of nNOS neurons.

## Graphical Abstract

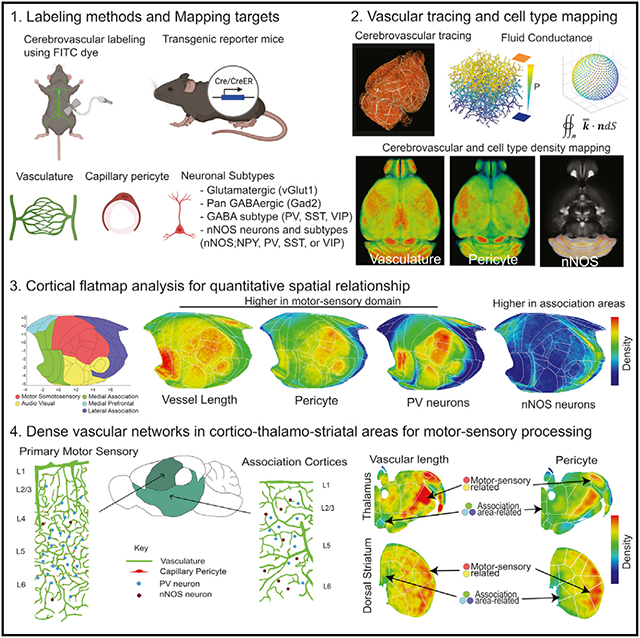

## INTRODUCTION

The brain meets its uniquely high metabolic demand through an intricate web of vascular and mural cells that dynamically supply blood and clear metabolic waste (Hartmann et al., 2015; Sweeney et al., 2016; Vergara et al., 2019; Zhao et al., 2015). Pericytes, a key mural cell type, wrap around microvessels and have been proposed to regulate blood flow and vascular permeability (Hall et al., 2014; Hartmann et al., 2021; Hill et al., 2015; Nikolakopoulou et al., 2019; Sweeney et al., 2016). Neuronal activity can also regulate local energy supply by controlling vascular diameter directly or indirectly (via astrocytes), which is referred to as neurovascular coupling (Attwell et al., 2010; Cauli and Hamel, 2010; Kaplan et al., 2020; Lecrux et al., 2011; Schaeffer and Iadecola, 2021). Neuronal health and function critically depend on efficient vascular support (Hall et al., 2012; Vergara et al., 2019). Impairment of the cerebrovasculature, pericytes, and neurovascular coupling has been widely implicated in many neurological disorders, such as stroke and neurodegenerative disorders, and even neurodevelopmental disorders (Ouellette et al., 2020; Zhao et al., 2015). Despite its significance, we have limited knowledge of the cellular architecture of the vasculature and pericytes, especially with respect to their quantitative relationship with neuronal cell types across the whole brain. This relationship is likely of critical importance for the heterogeneous coupling of neural activity to blood flow described across different brain regions (Devonshire et al., 2012; Huo et al., 2014; Shih et al., 2009; Zhang et al., 2019).

Generation of action potentials and synaptic transmission are energetically demanding (Harris et al., 2012; Howarth et al., 2012), and, accordingly, brain energy consumption has been proposed to be linearly correlated with the number of neurons in the brain across different animal species, including humans (Herculano-Houzel, 2011). However, neurons comprise highly distinct subtypes with different morphological, electrophysiological, and molecular characteristics (Kepecs and Fishell, 2014; Tasic et al., 2018). For example, the major classes of GABAergic neurons in the cortex include neurons expressing parvalbumin (PV), somatostatin (SST), and vasoactive intestinal peptide (VIP), each of which make distinct synaptic connections with pyramidal neurons and each other (Kepecs and Fishell, 2014). These neuronal cell types are expressed at different densities across cortical areas; PV interneurons have high density in sensory cortices and low density in association cortices, whereas SST neurons show the opposite density pattern in mice (Kim et al., 2017). Different neuronal subtypes also have differential energy demands and regulation. For instance, the fast-spiking PV neurons are among the neuronal types with the highest energy demand (Inan et al., 2016). Another neuronal type, neurons expressing neuronal nitric oxide synthase (nNOS), can actively regulate blood supply by causing vasodilation (Echagarruga et al., 2020; Krawchuk et al., 2020; Lee et al., 2020). These data suggest that determining specific spatial relationships between neuronal cell types and the vascular network is critically important for understanding differences among brain regions in terms of energy demand and the mechanism of distinct blood flow regulation across different brain regions.

To address the relationship between cerebrovasculature and the aforementioned cell types, we have devised high-resolution 3D mapping methods to derive a cellular architecture atlas containing cerebrovasculature, capillary pericytes, and several major neuronal cell types, including PV interneurons and vasomotor nNOS neurons, in the adult mouse brain. Our data resource allowed us to uncover key organizational principles of the brain, including a dense cerebrovascular network in primary motor sensory cortical areas and related thalamic and dorsal striatal areas and a positive correlation between vascular and capillary pericyte densities with energy-demanding PV interneurons in the isocortex.

## RESULTS

### Comprehensive vascular mapping in the intact mouse brain

**Figure 1.**
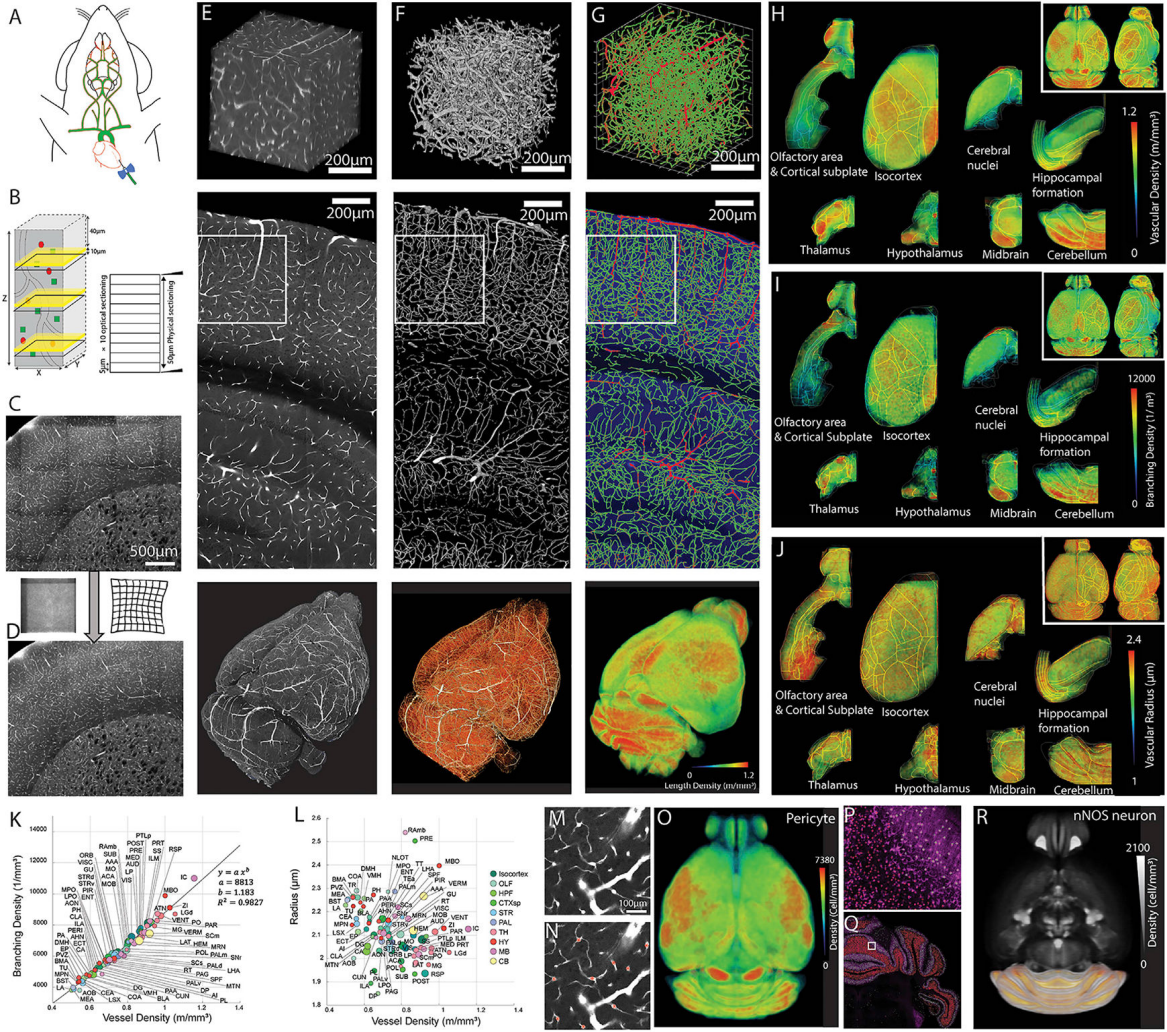
High-resolution 3D mapping of the cerebrovasculature, pericytes, and neuronal cell types (A) Fluorescent dye (FITC)-conjugated albumin gel perfusing the mouse brain through the heart to label cerebrovasculature. (B) Combination of physical and optical sectioning to achieve lossless imaging of a sample. (C and D) Stitching with optical aberration and tile line correction (D) from uncorrected images (C). (E–G) Example outputs from each stage of the analysis pipeline. Top row: 100-μm-thick 3D volume from the white boxed areas from the center row. Center row: an example coronal section. Bottom row: whole-brain results. (E) The raw image volume of FITC-labeled vasculature. (F) The binarized vasculature. (G) The traced vasculature. Large (radius > 5 μm) and small vessels are colored red and green, respectively. The bottom image shows the vasculature density. (H–J) The averaged vasculature length density (H), branching density (I), and radii (J) from four C57bl/6 mouse brains. (K and L). The correlation between vessel density and branching density (K) and the correlation between vessel density and the averaged radius (L). The size of each ROI is displayed according to the relative volume of the area. See Table S1 for abbreviations. (M–O). Pericyte density mapping. Shown is an example of tdTomato labeling from PDGFRβ-Cre; Ai14 mice (M), a pericyte detection algorithm (red stars, N), and brain-wide pericyte density (n = 10 brains) (O). (P–R) Pan nNOS neuronal mapping using nNOS-CreER; Ai14 mice (n = 10 brains). Shown is artificial intelligence (AI)-based detection of nNOS cells with two distinct shapes (green and red crosses) in the cerebellum (P, from the white box in Q) and brain-wide nNOS density (R).

Our first goal was to map spatial arrangements of the cerebrovasculature in the whole intact mouse brain to understand the anatomical variation in the vascular network. We filled microvessels from 2-month-old C57BL/6 mice with a cardiac perfusion of fluorescein isothiocyanate (FITC)-conjugated albumin gel (Figure 1A; Blinder et al., 2013; Tsai et al., 2009) and used serial two-photon tomography (STPT) imaging in combination with two-photon optical scans and serial sectioning to image the whole mouse brain at 1 × 1 × 5 μm (x, y, z; medial-lateral, dorsal-ventral, rostral-caudal) (Figure 1B). We also developed a stitching algorithm to correct optical aberrations, bleaching in overlapped tile areas, and z stack alignments (Figures 1C and 1D; Figure S1). We also developed a computational pipeline to quantitatively analyze cerebrovascular arrangement across the whole brain (Figures 1E-1G; see STAR Methods for more details). Individual brains were then registered to the Allen Common Coordinate Framework (Allen CCF) (Figures 1H-1L; Table S1; Video S1; Wang et al., 2020). We implemented an additional quality control step to reject data from areas with potentially incomplete labeling or imaging artifacts (Figure S1). We also confirmed that our approach closely reflects vasculature structure, including diameter, *in vivo* by directly comparing STPT results with *in vivo* two-photon measurements of the same vasculature acquired in the same mice (Figure S2).

We then focused on mapping the density distribution of capillary pericytes, using transgenic labeling of platelet-derived growth factor receptor β (PDGFRβ), and nNOS-expressing neurons as vasomodulatory cell types and major cortical cell types (pan glutamatergic, pan GABAergic, PV, SST, and VIP neurons) with different energy demands. We employed cell-type-specific reporter mice to genetically label 11 target cell types (Table S2). Then we developed deep learning algorithms to specifically count capillary pericytes and detect neurons, including densely packed nNOS neurons, in the cerebellum (Figures 1M-1N and 1P-1Q; Figure S3; Table S3; see STAR Methods for more details). Detected signals were then registered onto the Allen CCF to quantify the 3D density of the target cell type distribution across the whole brain (Figures 1O and 1R; Tables S3, S4, S5, and S6; Videos S2, S3, and S4; Kim et al., 2017; Wang et al., 2020).

### Primary motor sensory cortices show denser vasculature than association cortices

**Figure 2.**
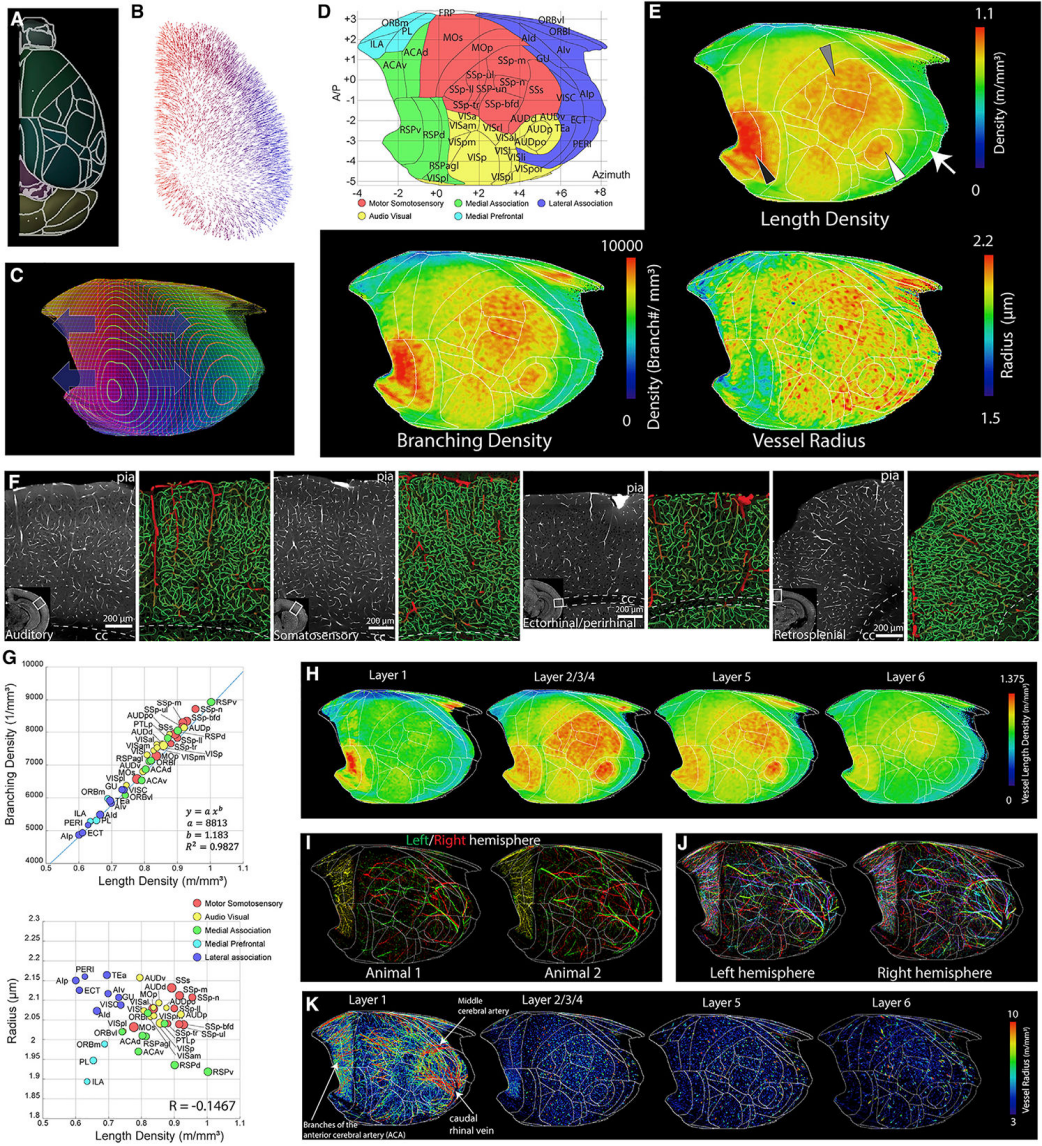
Heterogeneous vascular arrangements in the isocortex (A–C) Creating an isocortical flatmap. (A) Anatomical border lines of the Allen CCF. (B) Gradient vectors from solving the Laplace equation by setting cortical layer 1 and layer 6 as endpoints. (C) The flattened projected profile. (D) The cortical flatmap with Allen CCF border lines. y axis: bregma anterior-posterior (AP) coordinates; x axis, azimuth coordinate representing the physical distance by tracing the cortical surface on the coronal cut. (E) The averaged vasculature length and branching density as well as vessel radius plotted onto the cortical flatmap. Note the high density of vasculature in the SS (gray arrowhead), auditory (white arrowhead), and retrosplenial (black arrowhead) cortices; there is a low density in the lateral association cortex (white arrow). (F) Examples of cortical areas with different vasculature structures. Large (radius > 5 μm) and small vessels are colored red and green, respectively. (G) Correlation between average vessel density and branching density (top) or average radius (bottom) in the isocortex. See Table S1 for abbreviations. (H) Cortical layer-specific max projection of vasculature length density. (I) Large surface vessels from the left (green) and right (red) hemisphere from two different animals. (J) Large surface vessels from 4 different brains with different colors (red, green, blue, and cyan) in each hemisphere. (K) Large surface and P vessels (8 hemispheres from 4 animals) in a cortical layer-specific flatmap based on their radius.

We focused our analysis on the isocortex. To examine the spatial distribution intuitively while maintaining high-resolution information, we devised an isocortical flatmap based on Laplace’s equation (Figures 2A-2D; see STAR Methods for more details). We grouped isocortical areas into 5 subregions based on their anatomical connectivity and cell type composition: motor somatosensory, audio visual, medial association, medial prefrontal, and lateral association (Kim et al., 2017; Zingg et al., 2014; Figure 2D). When averaged vessel length density is plotted in the cortical flatmap, primary motor and sensory (auditory, somatosensory, and visual) cortices show overall higher vascular densities than association areas (medial prefrontal, medial, and lateral association) (Figures 2D-2H). For example, densely vascularized areas are tightly aligned with anatomical borders in the somatosensory (SS) and primary auditory (AUDp) cortices (Figure 2E, gray and white arrowheads). One notable exception is the ventral retrosplenial cortex (RSPv), which shows remarkably high vascular density compared with other cortical areas (Figure 2E, black arrowhead). Although the RSPv was included as a part of the medial association group, this area receives spatial navigation information from the dorsal subiculum (Zingg et al., 2014) and, thus, can be considered a sensory processing region. Cortical layer-specific maximum projection of the length density shows that sharp boundaries between cortical areas are strongly driven by layer 2/3/4 (specifically layer 4 for primary sensory regions) vascular distribution (Figure 2H).

Next we analyzed the vascular branching density and radius. Branching density closely followed the pattern of the vessel length density (Figures 2E-2G). In contrast, the average vessel radius did not show a significant correlation with the vessel length density (Figures 2E-2G). However, plotting vessel radius against vessel length densities unveiled distinct patterns between the five cortical groups. For instance, motor sensory areas showed overall high vascular density and radii, whereas the medial prefrontal group was low in both measurements (Figure 2G). The lateral association group showed a high vascular radius with relatively lower vascular density, and the medial association regions showed the opposite pattern (Figure 2G). the RSPv showed an overall low average vascular radius despite a high vascular length density (Figures 2E-2G).

We then examined whether a relatively large vasculature (radius > 3 μm) also showed regional variabilities. First we examined whether surface vasculature is stereotypically organized between samples and even between hemispheres of the same brain. We observed that the position of large surface vasculature differs considerably between brains and even between hemispheres from the same brain (Figures 2I and 2J). When we plotted the distribution of the large vessels from 8 hemispheres from 4 animals in our layer-specific flatmap, the layer 1 map including the surface vessels showed that the large vessels, including the middle cerebral artery (MCA), closely surround the somatosensory (SS) area (Figure 2K, layer 1).A high density of large penetrating vasculature was clearly observed in the primary motor sensory areas and the RSPv in layers 2–4, with a gradual decrease in deeper layers (Figure 2K).

These data provide strong evidence that cortical vascularization is not uniform but distinctly organized in functionally different cortical areas.

### Structure-based simulation reveals regional heterogeneity of microvascular directionality and fluid conductance

**Figure 3.**
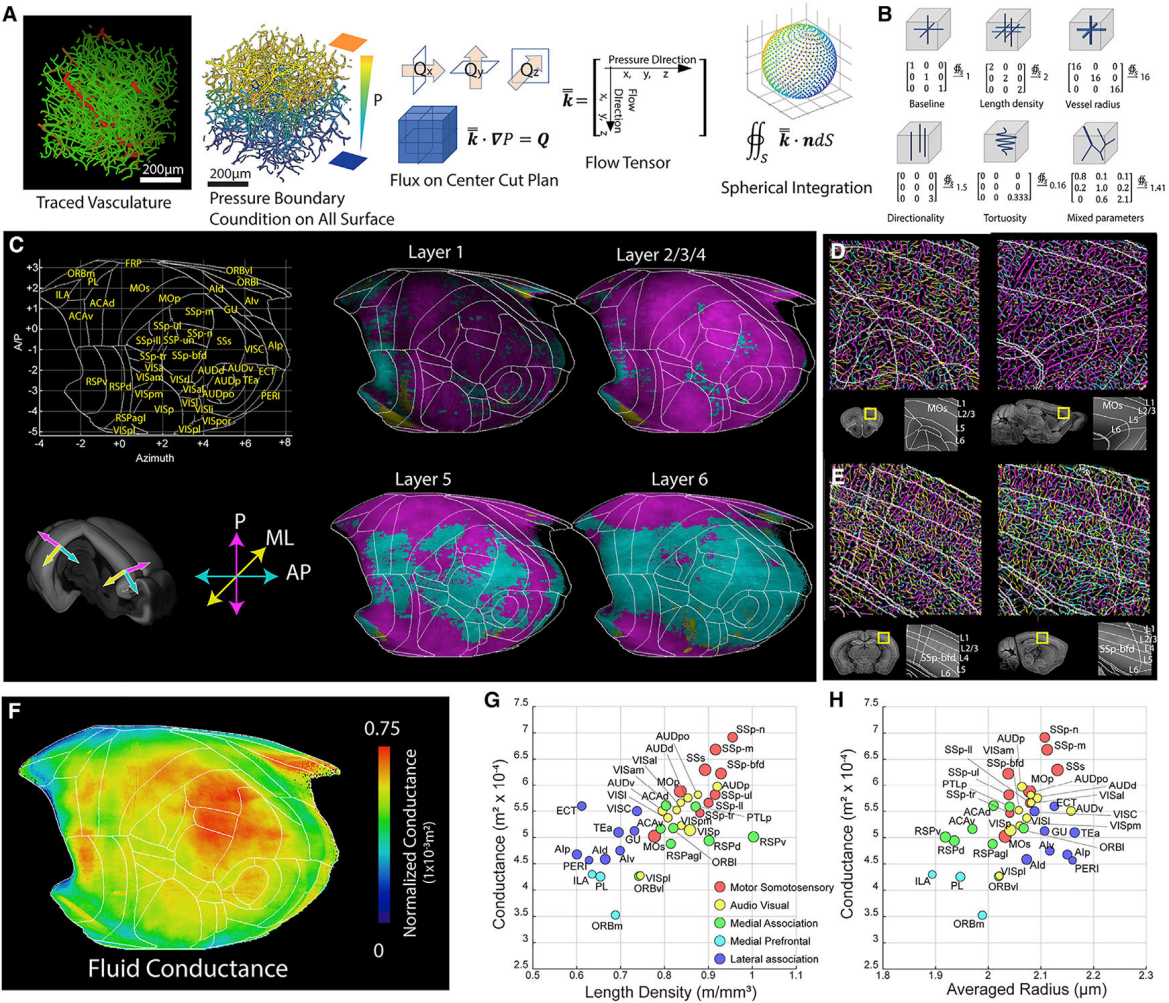
Anisotropy of the cerebral microvascular network and its fluid conductance (A) Flow chart of the fluid conductance simulation. From left to right: original traced data (red for large vessels with radius >5 μm and green for small vessels), applying a pressure profile on the surface of the control volume with a gradient profile and solving the coincide flux equation set for the flow tensor, the annotation rule of the flow tensor, and the sampling dots of the numerical spherical integration. l, perfusing length; *P*, pressure; *Q*, fluid flux; *R,* resistance; *μ*, viscosity; *k*, fluid conductance; Δ, changing of the quantity; ·, dot product; ∇, gradient; *∯s*, spherical integral; *S*, spherical surface; *n*, normal direction; bold font, vector; double top bar, tensor. (B) Examples illustrating how the structure of the vasculature network affects the flow tensor. (C–E) Microvessel anisotropy measurement in the isocortex. (C) Microvessel directionality in cortical layers. Only the dominant direction is displayed for simplicity. Microvessels are colored based on their orientation: magenta for penetrating (P), cyan for anterior-posterior (AP), and yellow for medial-lateral (ML). Shown are examples from the secondary motor cortex (MOs; D) and primary SS barrel field (SSp-bfd; E) cortices. Left: coronal view. Right: sagittal view. The color of individual vessels in the top panel (full-resolution images of the yellow boxed areas in the bottom left panel) represents three directions as in (C). White lines in the top panel denote anatomical annotations from the bottom right. Note the differences in dominant vessel directions based on brain regions and cortical layers. (F) Fluid conductance results in the cortical flatmap. (G and H) Relationship between fluid conductance and vessel length density (G) or average radius (H). See Table S7 for full data and abbreviations.

To examine the link between microvessel structure and its influence on blood perfusion in the brain, we applied a mathematical approach to estimate the fluid conductance and directionality of the microvascular network (Figures 3A and 3B; Video S5; Table S7).

We first examined how the geometry of microvascular networks can influence the directionality of blood flow by calculating the microvessel anisotropy using the tensor in the isocortex (Figure 3C). We used three axes according to the cortical column direction: the penetrating (P) axis along the cortical column as a main blood input direction from surface vessels and the anterior-posterior (AP) and medial-lateral (ML) axes as two vascular communication directions within areas (Figure 3C). Our analysis showed that microvessels oriented along the P axis dominated in the anterior (e.g., prefrontal and motor area) and posterior cortical areas (e.g., visual area), whereas mid-cortical areas (e.g., the SS area) showed vasculature orientation along the P and AP axes dominating the superficial layer and the deep layers, respectively (Figure 3C). For instance, the secondary motor cortex (MOs) shows dominant P axis vasculature (magenta), whereas the primary SS barrel field (SSp-bfd) shows a clear switch to AP axis (cyan) vasculature, preferentially between layers 4–6 (Figures 3D and 3E). This result suggests that mid-cortical areas containing many primary motor sensory areas have a high degree of AP vascular communication in deep layers to facilitate blood perfusion across these hypervascularized areas.

Next we applied the fluid conductance measurement across the isocortex and plotted the result in the cortical flatmap (Figure 3F). Overall, motor sensory groups showed higher fluid conductance than association groups in correlation with vascular density (Figures 3F-3H; Table S7). Noticeable exceptions include the RSPv with relatively low fluid conductance despite having the highest cortical vessel density because of small vessel radius and a few lateral association areas (e.g., the ectorhinal cortex [ECT]) which has relatively high fluid conductance because of large vessel radius (Figures 3G and 3H).

Our data suggest that microvessels in primary motor sensory cortices are structured to provide a high degree of blood perfusion compared with other cortical areas.

### Pericyte and nNOS neuron density mapping reveals differential vasoregulation between cortical areas

Pericytes, a mural cell type, are primarily located within the microvasculature and play important roles to regulate microvascular integrity and permeability (Attwell et al., 2016; Bennett and Kim, 2021; Hartmann et al., 2021; Nelson et al., 2020; Nikolakopoulou et al., 2019).

**Figure 4.**
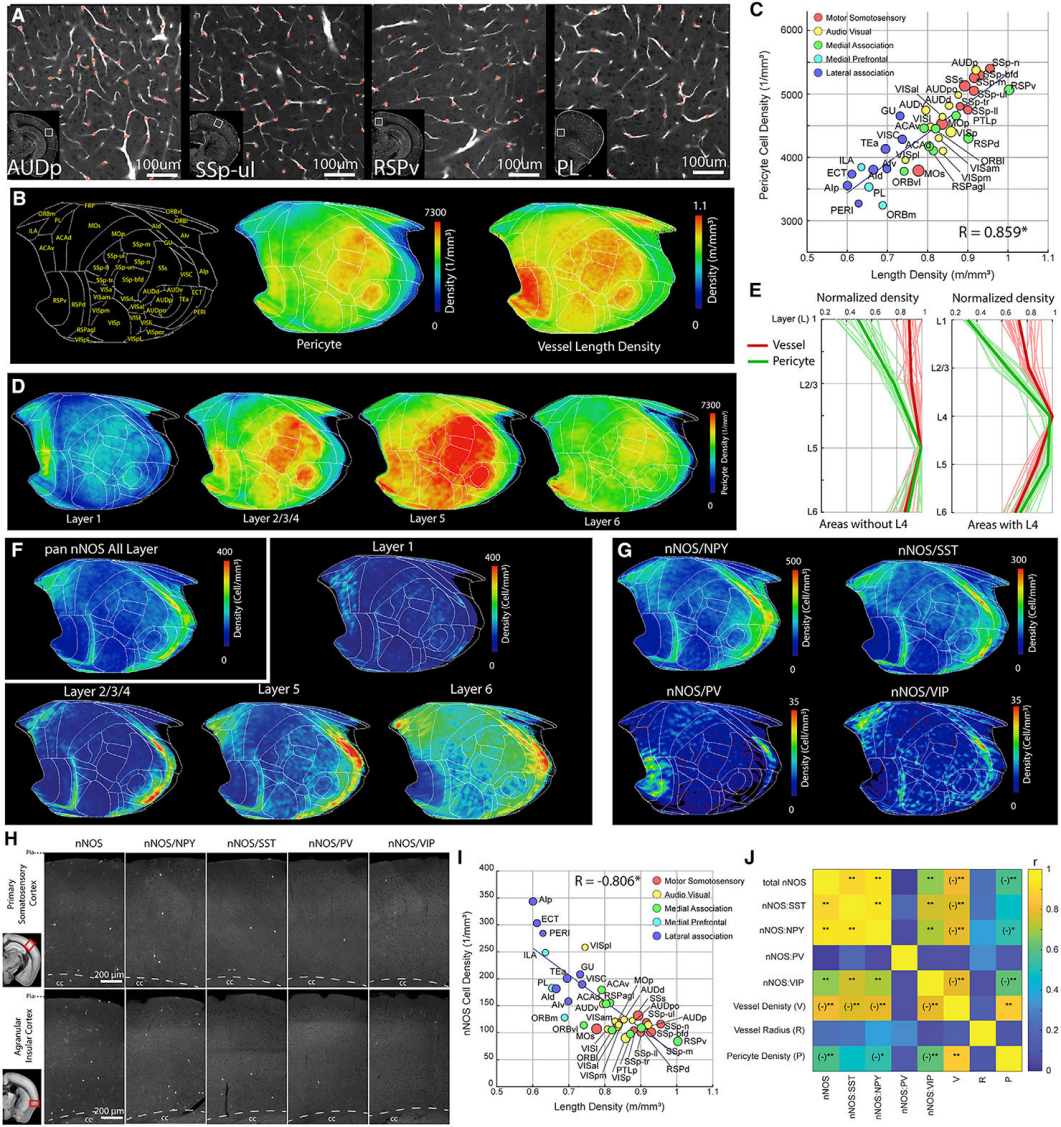
Cortical pericyte and nNOS neuron densities display opposite correlation with vascular density (A) Example images of areas showing variability in pericyte density from PDGFRβ-Cre; Ai14 mice with the pericyte detection algorithm (red stars). (B) Cortical flatmap of averaged pericyte density across the isocortex in comparison with the vessel length density. (C) Scatterplot demonstrating significantly positive correlation between pericyte density and vascular length density in isocortical regions (R = 0.859, p = 1.86 × 10^−12^). See Table S4 for abbreviations. (D) Layer-specific pericyte distribution. (E) Relative pericyte (green) and vessel (red) density (normalized against maximum value within the area) in cortical regions without (left) or with layer 4 (right). (F and G). Averaged density of pan nNOS neurons (F) or nNOS subtypes (G) on the cortical flatmap. Note the higher nNOS density in the medial prefrontal and lateral association areas. (H) Representative STPT images of nNOS cell types from the primary SS cortex and the agranular insular cortex. (I) Scatterplot showing significant negative correlation between total nNOS density and vessel length density in the isocortex (R = −0.806, p = 6.9 × 10^−7^). (J) Correlation matrix between nNOS cell types and vascular/pericyte measurements. *p < 0.05, **p < 0.005 after Bonferroni correction. (−) denotes negative correlation.

We wanted to determine whether capillary pericyte distribution shows distinct densities across brain areas in support of local brain functions in a similar fashion to cortical vasculature. We found that capillary pericyte density across cortical areas showed a distribution pattern similar to the vascular density (Figures 4A-4C; Tables S3; Video S2). Overall, primary sensory areas as well as the RSPv showed higher pericyte densities than association groups (Figures 4A-4C). A very strong positive correlation between capillary pericyte and vascular densities suggests that the number of pericytes per length of microvessel is overall constant across different cortical areas (Figure 4C). However, when we examined layer-specific differences in the density of capillary pericytes and vasculature, we observed that relative pericyte density as well as the ratio between capillary pericyte and vascular densities were highest in layer 5 and lowest in layer 1 (Figures 4D and 4E; Table S4). This suggests that layer 5, characterized by its large pyramidal neurons, may require higher capillary pericyte coverage per microvessel to finely tune the regulation of blood flow compared with other cortical layers.

Next we examined the relationship between the cerebrovascular network with capillary pericytes and the distribution of cortical nNOS-expressing neurons, whose activity has been shown to cause vasodilation (Echagarruga et al., 2020; Krawchuk et al., 2020; Lee et al., 2020). Based on our results so far, our expectation was that the density distribution of nNOS neurons may follow the distinct patterns of vasculature and pericyte densities in providing more robust blood flow support for the motor sensory versus prefrontal association cortical areas. Although we did observe up to 2-fold differences in nNOS neuronal density across isocortical regions (Figure 4F; Table S5; Video S3), nNOS neuron density was higher in the association cortical areas compared with the motor sensory areas—the opposite pattern than that of the vascular and pericyte densities (Figures 4F and 1H-1J). We also noted that the highest density of nNOS neurons was found in layer 6 in all cortical areas compared with the highest densities of vasculature and pericytes in layer 5 (Figure 4F). For nNOS subtypes, the nNOS/neuropeptide Y (NPY), nNOS/SST, and nNOS/VIP subtypes showed similar density patterns as the pan nNOS neurons (Figure 4G). In contrast, nNOS/PV neurons, despite having much lower density, showed relatively higher expression in the RSPv (Figure 4G), suggesting a subtype-specific role in this cortical area. The contrasting nNOS distribution is also evident from a correlation analysis of the relationship between nNOS neuronal densities and vascular density across different areas, which revealed a significant negative correlation in the isocortex that is true for all subtypes except nNOS/PV (Figures 4I and 4J). Similarly, nNOS neurons, including the nNOS/NPY and nNOS/VIP subtypes, showed significant negative correlation with pericyte density (Figure 4J). In contrast, nNOS neurons and all of their subtypes did not show any correlation with average vasculature radius (Figure 4J). These data show a surprisingly distinct distribution of nNOS neurons across cortical areas, with overall stronger nNOS-based vasomotor regulation in association cortices than in primary motor sensory cortices.

### Cortical PV interneurons and glutamatergic neurons show positive correlation with the vascular network

**Figure 5.**
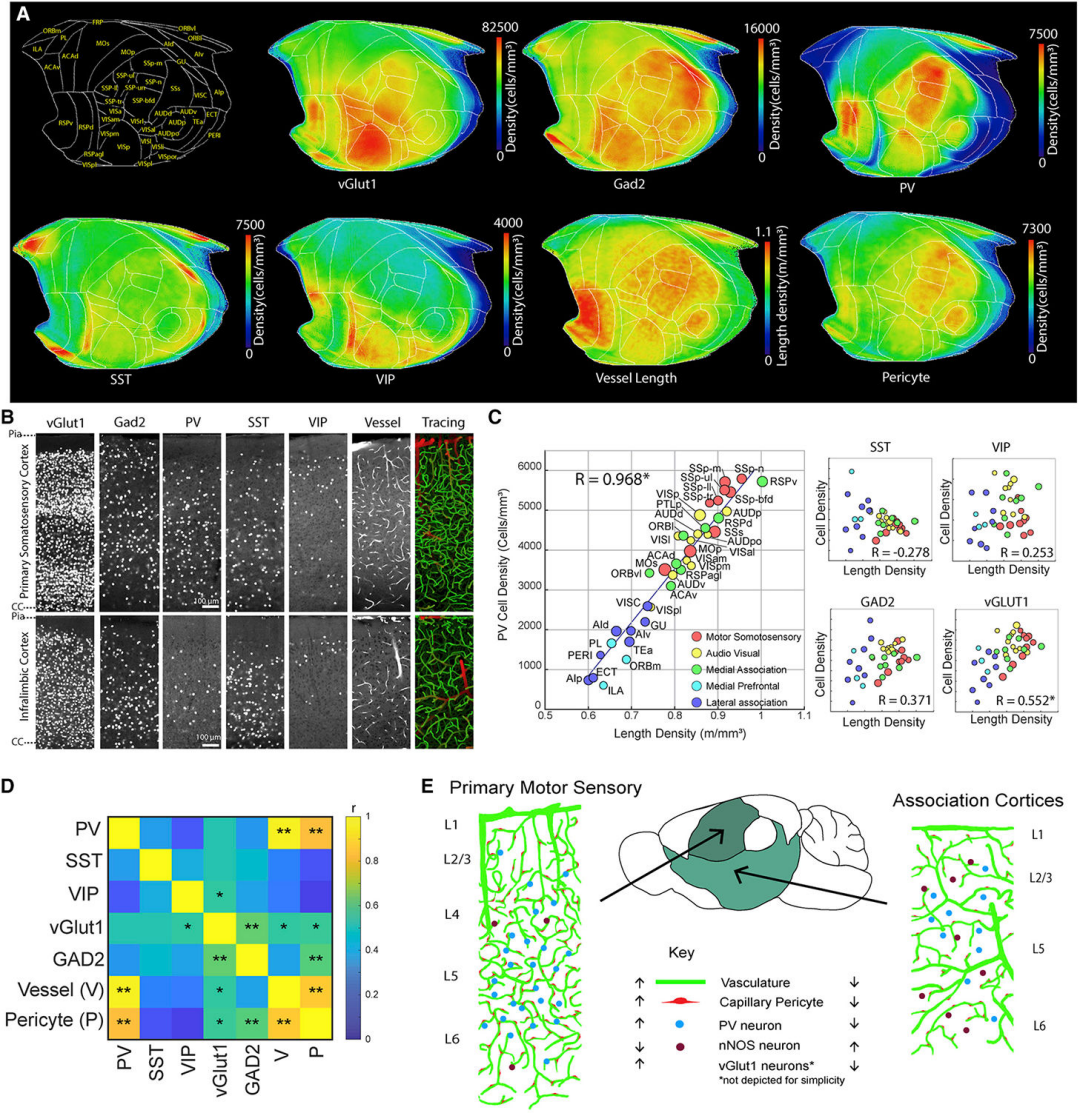
Cortical PV^+^ and vGlut1^+^ neurons positively correlated with vasculature density (A) Cortical flatmap showing density distributions of neuronal subtypes as well as vessel length and pericyte densities. (B) Examples of neuronal cell types and the vasculature and its tracing result (large vessels, red; microvasculature, green) from the densely vascularized primary SS and sparsely vascularized infralimbic cortices. (C) Correlation between vascular density and neuronal subtypes. Note the very strong positive correlation with PV density (R = 0.968, p = 8.5 × 10^−22^) and positive correlation with vGlut1 excitatory neuronal density (R = 0.552, p = 5.9 × 10^−3^). (D) Correlation matrix between neuronal subtypes, vessel length density, and pericyte density. *p < 0.05, **p < 0.005 after Bonferroni correction. (E) Cortical organization of the vascular/pericyte network and neuronal cell types. Primary motor sensory cortices are characterized by relatively high density of vessels, pericytes, PV interneurons, and vGlut1 excitatory neurons and low density of nNOS neurons. In contrast, association cortices show the opposite pattern.

Glutamatergic and GABAergic neuronal cell types have different energy consumption and metabolic costs (Buzsáki et al., 2007). We examined whether glutamatergic neurons and specific GABAergic neuronal subtypes show any significant correlation with vascular and capillary pericyte distribution in the isocortex (Figure 5; Table S6; Video S4). Density plotting using our isocortical flatmap allowed us to visualize distinct neuronal cell type distributions and localizations across the isocortex, revealing a clear pattern compared with the vessel length and pericyte densities (Figure 5A). First, pan-glutamatergic neurons (vGlut1^+^), but not pan GABAergic (Gad2^+^) neurons, showed modest but significant positive correlation with vascular and pericyte density (Figures 5A-5D). However, among different GABAergic cell types, PV^+^ interneurons showed a strikingly strong positive correlation with the vascular length density, whereas the other interneuron subtypes (SST^+^ and VIP^+^) did not show a significant correlation with vascular density (Figure 5C). All motor sensory groups and the RSPv showed high PV^+^ interneuron density comparable with the vascular density (Figures 5A and 5C). As expected, the pericyte distribution followed similar correlation patterns as the vessel length density with all neuronal subtypes studied (Figure 5D). Importantly, cortical PV neurons are involved in generation of gamma band oscillations (Cardin et al., 2009; Sohal et al., 2009; Takada et al., 2014), which are linked to increased vasodilation and blood flow in the brain (Drew et al., 2020). Thus, our results suggest that correlated vascular, pericyte, and PV^+^ interneuron densities may act to support local gamma-band oscillation neural activity during rapid signal processing in sensory cortices.

Our data suggest that the isocortex in mice is composed of two domains with distinct vascular/pericyte and neuronal cell type composition: (1) the primary motor sensory domain (motor, SS, audio, and visual cortices and the RSPv) with a high density of vessels and pericytes positively correlated to the density of PV^+^ inhibitory neurons and, less prominently, vGlut1^+^ excitatory neurons but negatively correlated with the density of nNOS neurons and (2) the association domain (lateral, medial, and medial prefrontal), which comprises the opposite pattern of vasculature, pericytes, and neuronal density distribution (Figure 5E; Table S8).

### The motor sensory thalamo-cortico-striatal circuit shares high densities of vasculature and capillary pericytes

**Figure 6.**
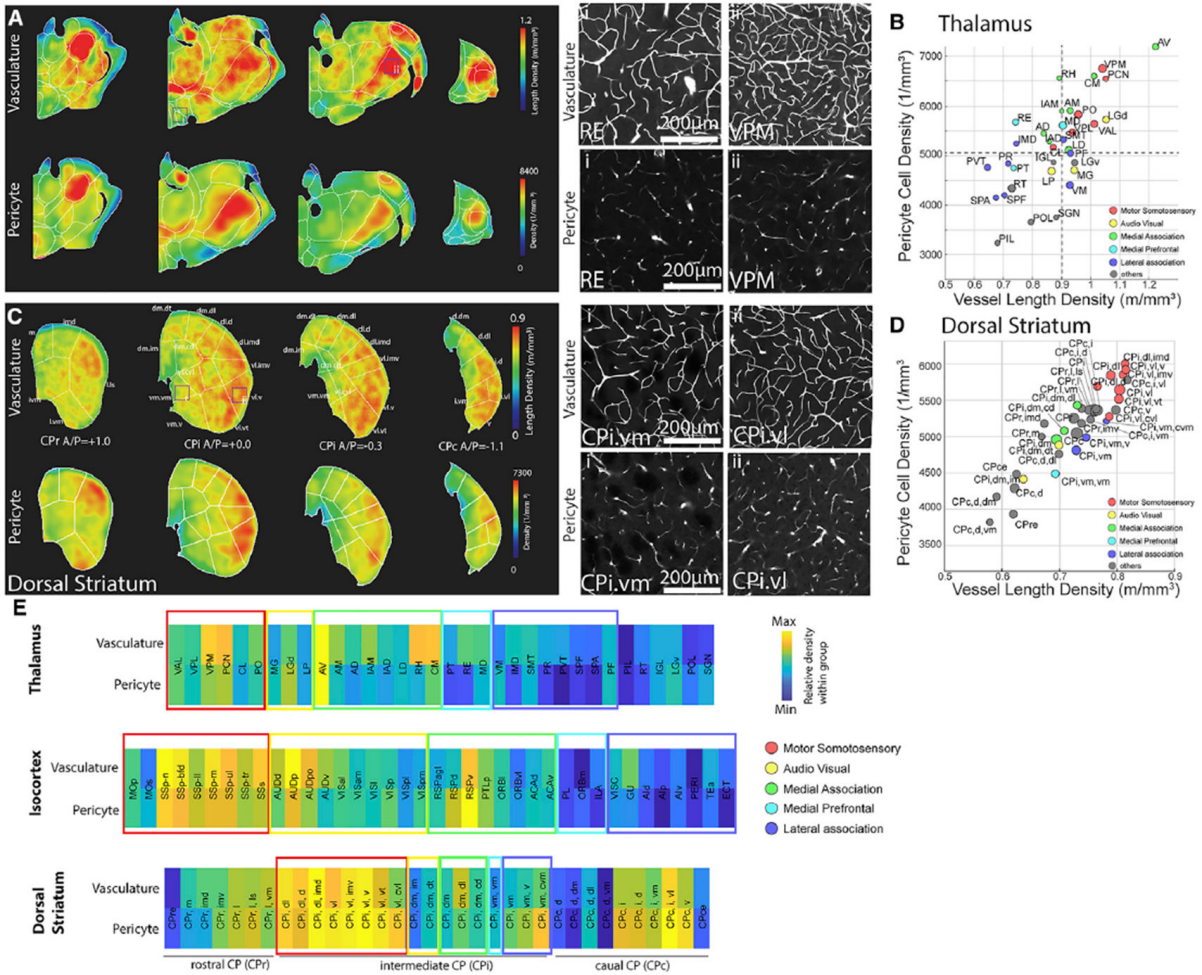
High density of vascular/pericyte network in the motor sensory thalamo-cortico-striatal pathway (A) Heatmap of vascular (top) and pericyte (bottom) densities in the thalamus (left side), with examples from the nucleus of reuniens (RE; low densities) and the ventral posteromedial thalamus (VPM; high densities) (right). (B) Density scatterplot of pericytes and vessel length densities. Colors of thalamic areas are assigned based on anatomical connectivity with specific cortical groups. (C) Heatmap of vascular (top) and pericyte (bottom) densities in the striatum (left), with examples from the intermediate CP ventral medial (CPi.vm; low densities) and intermediate CP ventral lateral (CPi.vl; high densities) (right). (D) Density scatterplot of pericytes and vessel length densities. The colors of striatal areas are the same as in (B). (E) Heatmap of vascular and pericyte densities normalized within anatomical areas. The colors of boxes represent cortical groups and their connected thalamocortical areas. Note that the motor sensory areas contain higher densities compared with association areas. See Table S9 for abbreviations.

Next we wanted to determine whether the high vascular and pericyte density specific to primary motor-sensory versus association cortices is also shared across thalamo-cortico-striatal pathways. We used thalamo-cortical and cortico-striatal connectivity datasets to identify clusters of thalamic and dorsal striatal areas that are well connected with the five cortical domains (Foster et al., 2021; Harris et al., 2019; Hintiryan et al., 2016; Hunnicutt et al., 2014). In the thalamus, sensory thalamic areas processing SS (e.g., ventral posteromedial thalamus [VPM]), auditory (e.g., medial geniculate complex [MG]), and visual (e.g., dorsal lateral geniculate complex [LGd]) information as well as the anteroventral nucleus of thalamus (AV), which is strongly connected with the RSPv, showed higher densities of vasculature and pericytes than non-sensory thalamic areas connected to medial prefrontal and other association cortical groups (e.g., nucleus of reuniens [RE]) (Figures 6A and 6B).

We then examined vascular and pericyte densities in subregions of the dorsal striatum (caudate putamen [CP]) using detailed anatomical segmentations established in the Allen CCF (Chon et al., 2019). The intermediate CP (Cpi) receives topographically segregated projections from cortical domains, whereas the rostral (CPr) and caudal (CPc) areas have intermixed projections from many cortical domains (Foster et al., 2021; Hintiryan et al., 2016). Our analysis revealed that Cpi areas receiving inputs from primary motor sensory cortices such as the Cpi ventral lateral (Cpi.vl) had relatively higher vascular and pericyte densities than the Cpi ventral medial (Cpi.vm) areas connected with medial prefrontal and lateral association cortical projections (Figures 6C and 6D; Table S9). Heatmap plots of relative vascular and pericyte densities with each anatomical region showed a clear pattern of primary motor sensory processing areas comprising overall higher vascular and pericyte densities compared with association areas throughout the thalamo-cortico-striatal pathways (Figure 6E).

### Data resources to further examine brain energy supply and regulation

Our brain-wide high-resolution vasculature and cell type mapping data open new opportunities to understand global and local energy supply and its regulation. For instance, we provide our brain-wide flow conductance simulation result and its relationship with capillary pericyte density and pericyte coverage (pericyte per vessel length density) as a resource to elucidate regional blood flow and vascular integrity (Figure S4). We noted that many hippocampal areas, including the dentate gyrus (DG) and the subiculum (SUB), showed low flow conductance, pericyte density, and pericyte coverage compared with other cortical and subcortical areas, which may confer regional vulnerability under pathological conditions (Figure S4; Table S7; Video S5; Ballinger et al., 2016; Sweeney et al., 2018).

We also include a freely downloadable simulation-ready dataset to model vascular flow in the whole brain as well as high-resolution raw images in a publicly available database. To facilitate ease of access and intuitive visualization to examine large-scale imaging datasets, we created a web-based resource (https://kimlab.io/brain-map/nvu/) that displays navigable z stacks of full-resolution images for our STPT datasets. This web-based resource also provides interactive 3D visualizations, allowing users to navigate our quantitative vascular and cell type measurements registered in the Allen CCF.

**Figure 7.**
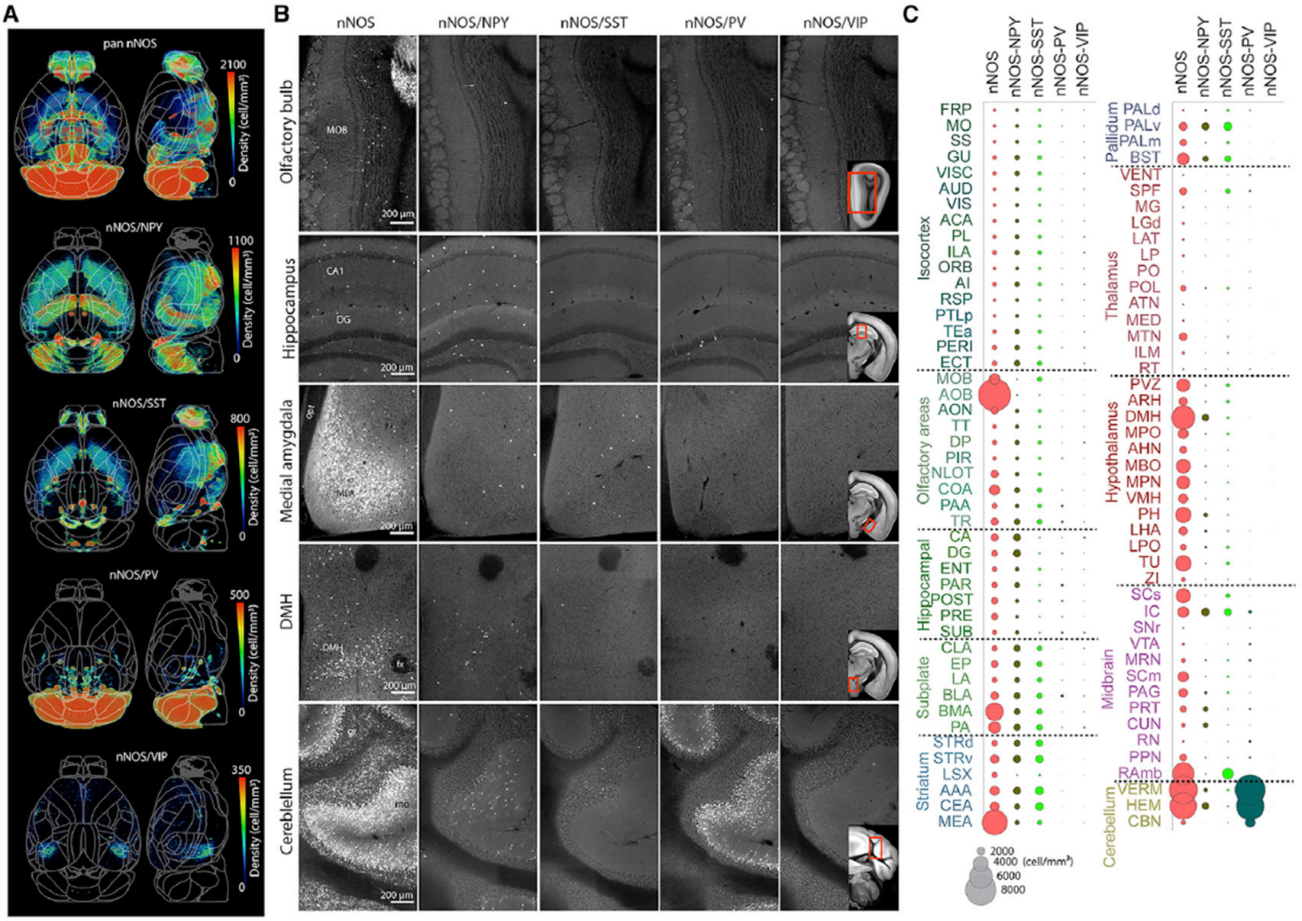
Brain-wide density map of nNOS neurons and their subtypes (A) Heatmaps demonstrating the distribution of total nNOS and nNOS subtype populations. See also Video S3 and Table S5. (B) Representative raw images of nNOS, nNOS/NPY, nNOS/SST, nNOS/PV, and nNOS/VIP neurons in the olfactory bulb, hippocampus, medial amygdala (MEA), dorsomedial hypothalamus (DMH), and cerebellum. Reference atlas images included in nNOS/VIP images show the area displayed for each region of interest. MOB, main olfactory bulb; CA1, Ammon’s horn; DG, dentate gyrus; opt, optic tract; fx, fornix; gr, granular; mo, molecular. (C). nNOS density by brain region for the total nNOS neurons and their subtypes. The size of a circle corresponds to density, as shown in the key at the bottom. See Table S5 for full names of abbreviations.

One notable cell type resource is the nNOS neuronal subtype brain-wide distribution data. In addition to cortical nNOS neurons, nNOS neurons in subcortical areas, including the cerebellum, also powerfully regulate neurovascular coupling (Du et al., 2015; Yang et al., 2003). Our results indicate that the total nNOS neuronal density is highest in the accessory olfactory bulb (AOB), followed by the cerebellum, medial amygdala (MEA), and dorsal medial hypothalamus (DMH) (Figures 7A-7C; Table S5). In contrast, the isocortex, hippocampus, and thalamus showed overall low nNOS neuronal density (Figure 7C). Of the nNOS subtypes, nNOS/NPY neurons represent the majority of nNOS subtypes in cerebral cortical, hippocampal, and cerebral nucleus areas (Figures 7A-7C). The nNOS/SST subtype showed overall similar density compared with the nNOS/NPY subtype except in hippocampal regions, which had noticeably low density (Figures 7A-7C). The nNOS/PV subtype showed overall low density across the whole brain, except for very high density in the cerebellum (Figures 7A-7C). The nNOS/VIP subtype had the lowest density compared with the other nNOS^+^ subtypes, with sparse expression in a few areas, such as the SUB of the hippocampus (Figures 7A-7C). Many amygdala and hypothalamic areas as well as the AOB showed high nNOS density that was not reflected in the nNOS interneuron subtype populations, suggesting that nNOS neurons in these areas may represent different nNOS subtypes (Chachlaki et al., 2017). Thus, the current brain-wide nNOS subtype mapping unveils region-specific distributions of the vasomotor neurons.

## DISCUSSION

The structural organization of regional vascular networks is crucial to support local brain function and may reflect susceptibility to different pathologies. Here we present cellular-resolution maps of cerebral vasculature, capillary pericytes, and neuronal subtypes in the mouse brain. Our cerebrovascular map, in combination with flow conductance simulations, reveals the organizational principles of microvessels and pericytes in relationships with several key neuronal cell types, highlighting regionally heterogenous vascular networks and potential differences in blood flow regulation across different brain regions.

### Cortical neuronal cell types and the vascular network

A prevailing theory of cortical organization is that the cortex is composed of repeating cortical columns with a common microcircuit motif (Douglas and Martin, 2004). However, this view has been challenged by recent data showing that different cortical domains show distinct cell type compositions and hemodynamic responses (Kim et al., 2017; Zhang et al., 2019). Results from the current study provide further evidence that vascular networks, including pericytes and vasomotor neurons, are organized in distinct spatial patterns to meet energy demands from motor sensory and association cortices (Howarth et al., 2012; Vergara et al., 2019).

Motor and sensory signals require precise temporal and spatial information processing in primary motor sensory cortices to perceive dynamic external signals and execute motor commands. Although previously included as a part of the association cortices, we consider the RSPv part of the sensory area because of its role in processing rapid navigational information from the dorsal SUB (Zingg et al., 2014). In contrast, association cortices integrate information from broader areas with slower temporal kinetics. We previously identified a higher density of PV neurons in sensory cortices compared with association cortices (Kim et al., 2017). Cortical PV neurons are fast-spiking interneurons that participate in generating gamma oscillations and are some of the most energy-demanding neurons (Cardin et al., 2009; Hu and Jonas, 2014; Inan et al., 2016; Kann, 2016; Takada et al., 2014). Thus, our current results suggest that a high density of microvessels and capillary pericytes in the sensory cortices provides an efficient energy support system for PV-dominated local circuits to accommodate high energy consumption and mediate local functional hyperemia for rapid sensory processing. In contrast, association cortices contain relatively high densities of nNOS neurons despite low vascular, capillary pericyte, and PV densities. Although nNOS interneurons represent only about 2% of cortical neurons, activation of nNOS neurons robustly dilates cerebral arterioles to generate increases in cerebral blood flow (Echagarruga et al., 2020; Krawchuk et al., 2020; Lee et al., 2020). Thus, the relatively higher density of nNOS neurons in association areas suggests that this cell type can exert more powerful vasodilation in larger areas to compensate for a lower vascular density in these highly cognitive areas.

We found that areas of the thalamus and the dorsal striatum heavily connected to motor-sensory cortices also contain high densities of vasculature and pericytes compared with thalamostriatal areas linked with association cortices. Thus, our data demonstrate that this dense network of vasculature and pericytes is conserved throughout neural circuits processing primary motor sensory information.

### Comprehensive data resources to understand relationships between brain regional vascular organization and energy homeostasis

Although recent approaches using light sheet microscopy to examine fine cerebrovascular structure have provided advantages in rapid data acquisition as well as 3D immunolabeling to mark different vascular compartments (Kirst et al., 2020; Todorov et al., 2020), the required tissue clearing methods can introduce microscopic volume distortions, which can lead to inconsistent measurements (Ji et al., 2021). Here we used STPT to visualize the whole cerebrovasculature at single-capillary resolution in intact mouse brains, revealing a cerebrovascular map that closely represents physiological conditions, as confirmed by *in vivo* two-photon microscopy. Thus, our dataset allows us to perform precise computational simulations to estimate fluid conductance based on structural arrangements of microvessels, including deep cortical layers as well as subcortical areas, which are hard to access with *in vivo* two-photon microscopy. Our high-resolution cerebrovascular maps combined with vasoregulatory cell types can provide a detailed structural basis of signals for functional neuroimaging modalities such as functional magnetic resonance imaging or newly emerging functional ultrasound imaging (Brunner et al., 2020; Lecrux et al., 2019).

Our study also presents a brain-wide quantitative capillary pericyte map. Previous functional studies identified that capillary pericytes actively regulate the diameter and permeability of microvessels (Hartmann et al., 2021, 2022; Nikolakopoulou et al., 2019; Rungta et al., 2018). Capillary dilation could have significant effects on blood flow to mediate functional hyperemia (Pfeiffer et al., 2021; Schmid et al., 2017). Our results complement previous mechanistic studies of a defined anatomical area by providing capillary pericyte population density across the brain. We observed a strong positive relationship between capillary pericyte and vascular density in the cortex, suggesting that pericyte coverage per microvessel remains similar across different cortical areas in the normal adult mouse brain. We found the highest pericyte coverage per vascular length in layer 5 across all cortices. Because large pyramidal neurons in layer 5 act as main cortical output to the rest of the brain, the high density of pericytes may confer extra control over blood flow in this energy-demanding layer (Schmid et al., 2017). Our subcortical mapping results provide opportunities to investigate pericyte arrangements in largely understudied brain regions. For instance, thalamic areas have overall higher pericyte density compared with other areas (Figure S4). Interestingly, thalamic pericytes have shown resistance to disrupted PDGFRβ signaling, whereas cortical and striatal pericytes were more vulnerable (Nikolakopoulou et al., 2017). The combination of high density and cellular resilience may confer extra protection to maintain vascular integrity in the thalamus. Conversely, relatively low pericyte density in the hippocampal areas and association cortices can make these areas more vulnerable to pathological conditions (Montagne et al., 2015; Sengillo et al., 2013; Zhao et al., 2015).

Our nNOS results with added subtype specificity offer insights to understand nNOS subtype coverage across the whole brain. In contrast to well-studied cortical nNOS neurons, the functional and vasomotor characteristics of subcortical nNOS neurons is largely unknown. Previous studies have suggested that nNOS signaling in the cerebellum and the hypothalamus is linked to neurovascular coupling (Du et al., 2015; Yang et al., 2003). Our comprehensive nNOS and nNOS subtype maps can guide future research to determine which brain regions and nNOS subtypes need to be examined to establish a causal relationship between nNOS neuronal types and local hemodynamic response.

### Limitations of the study

Caveats of the current study include a lack of separate labeling for different vascular compartments (e.g., arteries versus veins) to understand blood flow direction and relative simple fluid conductance measurements without considering granule-like properties in red blood cells. Future studies using discrete vascular labeling and computational modeling considering additional information (e.g., blood pressure and viscosity) can help to gain a more complete understanding of brain blood perfusion and its change with additional risk factors, such as stroke (Balogh and Bagchi, 2019; Blinder et al., 2010). Another limitation of the current study is the lack of delineation for pericyte subtypes in vascular subregions (Grant et al., 2019; Hill et al., 2015). Future studies with a combination of markers (e.g., CD13, smooth muscle actin) in the same brain will help to classify and quantify these cell types in the precapillary arterioles, capillaries, and post-capillary venules in the whole brain.

Our quantitative information on cerebrovasculature and associated cell types establishes a platform for future studies to gain a deeper understanding of how energy demand and supply maintain balance in a normal brain from a cellular architectural perspective and how this homeostatic mechanism changes under pathological conditions.

## STAR★METHODS

### RESOURCE AVAILABILITY

#### Lead contact

Further information and requests for resources should be directed to and will be fulfilled by the lead contact, Yongsoo Kim (yuk17@psu.edu).

#### Materials availability

This study did not generate new unique reagents.

#### Data and code availability

Deposited data and codes are listed in the Key resources table. All dataset and codes can be used for non-profit research without any restriction. Any additional information required to reanalyze the data reported in this paper is available from the lead contact upon request.

### EXPERIMENTAL MODEL AND SUBJECT DETAILS

Animal experiments were approved by the Institutional Animal Care and Use Committee at Penn State University and Cold Spring Harbor Laboratory. For all genotypes in this study, both adult male and female mice were used, unless otherwise specified. Adult 2-month-old C57BL/6 mice were bred from C57BL/6 mice directly obtained from the Jackson Laboratory and used for vascular tracing experiments with FITC filling (n = 4). For pericyte specific experiments, male PDGFRβ-Cre mice (Kind gift from the Volkhard Lindner Lab) (Cuttler et al., 2011) were crossed with female Ai14 mice (Jax: Stock No: 007914) as previously described (Hartmann et al., 2015). These PDGFRβ-Cre;Ai14 mice exhibit PDGFRβ-driven tdTomato expression in two distinct vascular cell types, pericytes and vascular smooth muscle cells (vSMCs). For isocortical cell types, vGlut1-Cre (Jax: 023527) and Gad2-Cre (Jax: 010802) mice were crossed with Ai75 reporter mice (Jax: 025106). nNOS-CreER mice were used to label nNOS neurons (Jax: Stock No: 014541)(Taniguchi et al., 2011). After nNOS-CreER mice were crossed with Ai14 mice, the nNOS-CreER;Ai14 offspring were administered with an intraperitoneal (i.p.) tamoxifen (Sigma, cat.no. T5648-1G) injection (100mg/kg) at P16. Similarly, for nNOS-subtypes, nNOS-CreER mice were initially crossed with Ai65 mice (Jax; Stock No: 021875), which were further crossed with PV-flp (Jax Stock No: 022730), SST-flp (Jax Stock No: 028579), NPY-flp (Jax Stock No: 030211), or VIP-flp (Jax Stock No: 028578) mouse lines, to generate triple transgenic mice which allowed for tdTomato fluorescent labeling of nNOS expression within these interneuron populations. To allow for postnatal specific expression of tdTomato in nNOS+ subtype populations, tamoxifen injections dosed at 75mg/kg were given at P10, P12, and P14 timepoints. We used 10 animals for each PDGFRβ;Ai14, nNOS;Ai14, nNOS;VIP;Ai65, 9 animals for nNOS;NPY;Ai65 and nNOS;PV;Ai65, 8 animals for nNOS;SST;Ai65. For PV, SST, and VIP neurons, we re-registered previously collected data on to the Allen CCF (Kim et al., 2017). For glutamatergic and gabaergic neuronal populations, results were obtained from 7 animals (all males) for vGlut1;Ai75, and 9 animals (all males) for Gad2;Ai75. All animals were used once to generate data. We used tail genomic DNA with PCR for genotyping. Brain samples were collected at 2 months old age for all mouse lines.

### METHOD DETAILS

#### Perfusion and tissue processing for STPT imaging

Animals were deeply anesthetized with a ketamine-xylazine mixture (100 mg/kg ketamine, 10 mg/kg xylazine, i.p. injection) for both regular perfusion and vascular labeling. Transcardiac perfusion with a peristaltic pump (Ismatec, cat.no.: EW-78018-02) was used with 1× PBS followed by 4% paraformaldehyde at 0.3mLs/min, both injected through a small incision in the left ventricle, in order to wash out blood and allow for tissue fixation, respectively. Brains were dissected carefully in order to preserve all structures. For vessel labeling, transcardiac perfusion with a peristaltic pump (Welch, Model 3100) was used with 1× PBS followed by 4% paraformaldehyde at 0.3 mL/min, in order to wash out blood and for tissue fixation, respectively. To ensure that the large surface vessels would remain filled with the gel perfusate, the body of the mouse was tilted by 30° before gel perfusion (with the head tilted down), as previously described (Tsai et al., 2009). Following the fixative perfusion, the mouse was perfused at 0.6 mL/min with 5 mL of a 0.1% (w/v) fluorescein isothiocyanate (FITC) conjugated albumin (Sigma-Aldrich, cat.no.: A9771-1G) in a 2% (w/v) solution of porcine skin gelatin (Sigma-Aldrich, cat.no: G1890-500G) in 1× PBS. Immediately after perfusion, the heart, ascending and descending aorta as well as the superior vena cava, were all clamped with a hemostat (while the butterfly needle was simultaneously removed from the left ventricle). This served to prevent any pressure changes in or gel leakage from the brain vasculature. Next, the entire mouse body was submerged in an ice bath to rapidly solidify the gel in the vessels. Then, the head was fixed in 4% PFA for one week, followed by careful dissection of the brain to avoid damage to pial vessels. After fixation and dissection, the brain was placed in 0.05M PB until imaging. Any animals that had poor perfusion and/or possible air bubbles interfering with the gel perfusion were excluded from imaging and any further analysis.

#### Serial two photon tomography (STPT) imaging

Prior to imaging, the brain sample was embedded in oxidized agarose and cross-linked in 0.05M sodium borohydrate at 4°C for at least 2 days ahead of imaging (Kim et al., 2017; Newmaster et al., 2020). This procedure allows for seamless cutting of 50μm thick sections using a built-in vibratome, while also preventing any tearing of the brain surface. The embedded brain sample was then glued to the sample holder and fully submerged in 0.05M PB in an imaging chamber. For STPT imaging (TissueCyte 1000, TissueVision), we used 910nm excitation using a femtosecond laser (Coherent Ultra II) for all samples. Signals in the green and red spectrum were simultaneously collected using a 560 nm dichroic mirror (Chroma). For pericyte and neuronal subtypes, STPT imaging was conducted with 1 × 1 μm (*x,y*) resolution in every 50 μm (*z*), with the imaging plane set at 40μm deep from the surface, as previously described (Kim et al., 2017; Newmaster et al., 2020). For vascular imaging, optical imaging (5 μm z step, 10 steps to cover 50μm in *z*) was added in the imaging, producing 1 × 1 × 5 μm (*x,y,z*) resolution beginning at 20μm deep from the surface. Due to length of imaging time required for vascular imaging, each brain sample was imaged through multiple imaging runs to adjust the imaging window size in order to reduce overall imaging time.

#### STPT image reconstruction

To measure and correct an optical aberration from the objective lens (Figure S1), we imaged a 25 μm EM-grid (SPI supplies, cat.no.: 2145C) as a ground truth for spatial data (Han et al., 2018). We annotated all cross points of the grid and computed the B-spline transformation profile from the grid image to the orthogonal coordinate sets using ImageJ (Schneider et al., 2012). The pre-scripted program then corrected every image tile by calling the ImageJ deformation function using that profile. Afterwards, we used the entire set of imaged tiles (full mouse brain in this case) to map out the tile-wise illumination profile. The images were grouped according to the stage movement, which affects the photo-bleaching profile. The program avoids using pixels that are considered empty background or dura artifacts using preset thresholds. Using those averaged profile tiles, the program normalized all the tile images. Please note, this profile is unique for each sample. Finally, the program picked 16 equal-spacing subsampled coronal slices (out of the nearly 2,000) throughout the z stack and utilized ImageJ’s grid/collection stitching plugin to computationally stitch those 16 slides. The program then automatically performs z-stack alignment through combining the transformation profiles from center to outer edge according to the calculated pairwise shifting distance to perform the z-stack alignment. It used a tile-intensity weighted average to ensure the empty tiles did not contribute to the final profile. This approach significantly reduced the computational time and allowed parallelization with no communication overhead. After the aforementioned alignment, the program finally stitched the image set together. If the sample was imaged through multiple runs during imaging acquisition, the program also aligns and combines each of these image sets into one cohesive image stack.

#### Vessel digitization, tracing, and visualization

We started with interpolating the data into 1 × 1 × 1 μm resolution with cubic interpolation then subtracted the signal color channel (green) with the background color channel (red) to remove auto-fluorescent backgrounds. Next we performed a voxel binarization. The voxel with at least one of the following conditions passed as the foreground signal (vasculature), a. the voxel passed a fixed threshold (6× that of the non-empty space average) or b. passed a threshold (2.4× that of the non-empty space average) after subtracting a circular 35% local ranking filter. The binarized image was then skeletonized using 26-neighbor rule (Kollmannsberger et al., 2017). The code then reconnected lose ends that were within 10 μm distance and removed all the short stem/furs shorter than 50 μm, starting with the short ones and iterated until no more fur artifacts were found (Figure S1F). The threshold of 10 μm was chosen based on the FITC vessel labeling quality and stitching quality. Most of faulty disconnection is less than 5 μm. The 50 μm threshold was chosen based on the 1:10 aspect ratio for capillaries to be consider too stubby to be true. By using the binary image and the skeleton (center-line), the radius for each skeleton pixel can be measured. The code then grouped all the skeleton pixels into segments with the branching nodes, and all the segments shorter than 2× radius (or shorter than 10 μm) were further cleaned up with shortest graph path (Figure S1G). ROIs with poorly connected (<250 μm/node) were excluded in further analysis as shown Figure S1H. Many of brain stem regions do not pass this threshold due to imaging issues related to high myelination. Finally, the code documented and traced all the segments and nodes with their connectivity, length, averaged radius, and raw skeleton locations. The full pipeline here is programed to be fully automatic and the code was fully vectorized and parallelized with reasonable memory consumption per thread (~8GB).

For 3D voxel visual rendering, we used 20 × 20 × 20μm^3^ as the bin size for voxelization with dataset registered onto the Allen CCF. then the data array was processed with gaussian filter (σ = 2) to achieve local averaging. The transparency was set linearly between 85% and 100% (100% is fully transparent) respected to upper and lower threshold of the colormap. We used 3D rendering tool (Avizo, ThermoFisher) to illustrate the voxel data for the spatial distribution of our 3-D measurements (e.g., volumetric length density).

#### Fluid conductance simulation

The goal of calculating and visualizing a flow tensor is to illustrate how well fluid can flow through the local microvasculature of a given volume in a given direction. Since the direction distribution of the microvasculature can be anisotropic, the fluid flow can move with a direction that is different from the pressure gradient direction, thus making the flow in a tensor form. Such a tensor can illustrate the local microvascular performance and its directional characteristic.

The equation of flow tensor is given by:

$$\bar{\bar{k}}\cdot \nabla P=Q$$

or

$$\left[\begin{array}{ccc} k_{xx} & k_{yx} & k_{zx}\\ k_{xy} & k_{yy} & k_{zy}\\ k_{xz} & k_{yz} & k_{zz} \end{array}\right]\left[\begin{array}{c} P_{,x}\\ P_{,y}\\ P_{,z} \end{array}\right]=\left[\begin{array}{c} Q_{x}\\ Q_{y}\\ Q_{z} \end{array}\right]$$

where *k* is the flow tensor, *P* is the pressure, *Q* is the fluid flux, the subscript index is the Cartesian coordinate direction, and the comma is partial differentiation. We chose a size of 400 × 400 × 400 μm as the local representative control volume. We then probed the system with three ∇*P* that are the three unit-vectors in the Cartesian coordinate system. The pressure boundary condition with gradient profile was applied on all size surfaces of the cubical control volume, then the network flow profile was calculated by solving the system of equations of the Hagen–Poiseuille equation (with the viscosity set to unity for normalization) and conservation of flux. We chose the center cut plan to measure the directional flux and consequently, the flow conductance. To illustrate the vascular directionality of the isocortex, we projected the tensor onto penetrating, anterior-posterior, medial-lateral vectors according to their location within the isocortex using the equation $k_{pj}=|\bar{\bar{k}}\;\cdot n_{pj}|$, where subscript *pj* indicates the direction of the projecting vessels. To calculate overall fluid conductance in a given voxel (400 × 400 × 400 μm^3^), we integrated flow tensor through numerical approximation of two thousand points distributed by Fibonacci Sphere. The integrated results of voxels (20 μm spacing) were used for Figure 3F and Video S5.

#### Deep learning neural network (DLNN) pericyte counting

We used a deep learning neural network (DLNN) to detect and classify cells. Instead of using a fully convoluted neural network like Unet, we chose to use per-cell multi-resolution-hybrid ResNet classification with potential cell locations (He et al., 2016). This makes the AI compute time significantly shorter. The potential cell locations were identified with local maximum within a radius of r = 8 μm. The image around the potential cell locations was fed to the network with two different resolutions. One is 101 × 101 μm (101 × 101 pixel) and the other one is 501 × 501 μm (201 × 201 pixel). The two-window system allows the network to capture characteristics from two zoom scales simultaneously. In order to use global maximum at the end of the network, we stacked an empty (value zero) image onto the 3D direction of each image, which made them 101 × 101 × 2 and 201 × 201 × 2 pixels. We then assigned value 1 to the location of the potential cell, in this case, the center. At the end of the two networks of those two images, the intermediate images were flattened and concatenated into one. The classification was done with two bins, ‘pericytes’ and ‘everything else.’ The detailed schematic describing the network is in Figure S3.

We deployed two human annotators with the same training to annotate the data, and only used the mutually agreed data to train the AI to eliminate human error and bias. We used a strict set of criteria to include only capillary pericytes. Cells were counted only when the cell body was in the imaging plane and clear pericyte cell morphology could be detected. Cells associated with larger vessels, often with vascular smooth muscle morphology, were not counted to prevent the inclusion of erroneous cell types. We also excluded transitional cell types often referred to as either ensheathing pericytes or precapillary arteriolar smooth muscle cells, due to the controversy in the field as to whether this should be included as a pericyte subtype and lack of additional markers to distinguish different pericyte subtypes in our image dataset (Attwell et al., 2016; Hartmann et al., 2015). A total 12,000 potential cell locations from multiple anatomical regions across 4 different brains were annotated by both annotators. 90% of the data selected at random was used to train the AI and the remaining 10% was used for validation. The 90% of the data taken for training was further truncated down to 3,400 potential cell locations with half positive and half negative for training. The positive cell selections in the raw data were around 19.6% (annotator #1) to 21.1% (annotator #2). The validation set was not truncated to represent actual performance. The performance can be found in Table S3.

#### Deep learning neural network (DLNN) nNOS neuron counting

The morphology and size of tdTomato positive cells in the granular layer of the cerebellum from nNOS-CreER; Ai14 mice differs significantly from other tdTomato positive nNOS neurons in other brain regions. Thus, we developed new DLNN AI algorithms to consider not only cell morphology but also the location of cells by putting additional zoomed-out, low-resolution images of whole coronal sections. The network set-up is similar to the pericyte classification with one more image containing the coronal section with the cell location. The inputs are 101 × 101 μm (101 × 101 pixel), 501 × 501 μm (201 × 201 pixel), and the full frame low resolution 12 × 8 mm (201 × 201 pixels). Similar to the pericyte network, we made images 101 × 101 × 2, 202 × 202 × 2, and 202 × 202 × 2 pixels with the cell location marked as value 1. At the end, those three sub-networks were flattened and concatenated into one. The classification is done with three bins, nNOS neurons, cerebellar granular nNOS neurons, and everything else. One human user created 10,000 annotations from 5 pan nNOS and 5 nNOS subtype brains. 5,000 cells from 5 brains were initially used to train the AI. Another 5,000 cells from 5 new brains (one of each Cre mice, i.e. nNOS/nNOS-SST/nNOS-PV/nNOS-VIP/nNOS-NPY) were used to evaluate the AI performance. The AI reached an F1 score = 0.96, which is comparable to human performance. The details for the network are in Figure S3. The performance can be found in Table S3.

#### Isocortical flatmap

We started with Allen CCF annotation images to solve the Laplace equation by setting the surface of cortical layer 1 as potential ‘1,’ the surface of layer 6b as ‘0,’ and the surface of everything else as flux ‘0’ (Wang et al., 2020). We used the potential map to find the gradient direction as the projecting direction. The projection was first traced to the cortical surface and then flattened at the Anterior-Posterior (A-P) tangential plane, which later preserved the A-P coordinate on the flat map. The flattened map has the y axis mapped as the original A-P coordinate at the surface, and the x axis was adjusted to represent the surface arc (azimuth) length to the reference *X*-zero. The reference *X*-zero was defined on the cortical ridge in the dorsal direction (maximum *Y* point in 3D) with a straight cut in the A-P direction. Finally, the projection profile was saved at two resolutions, 10 × 10 × 10 μm^3^ and 20 × 20 × 20 μm^3^. We created a Matlab script that can map any signal (previously registered to the Allen CCF) into a 3D projected isocortical flatmap.

#### Conversion of 2D based counting to 3D cell density

STPT imaging has very accurate cutting and stage depth movement, which allows us to convert the 2D cell counting to 3D cell density. We used previously calculated 3D conversion factors for cytoplasmic (factor = 1.4) and nuclear signals (factor = 1.5) to generate density estimates of nNOS neurons and other neuronal cell type datasets (Kim et al., 2017). To estimate the 3D conversion factor for pericytes as we have done for Figure S1 from (Kim et al., 2017), we imaged one PDGFRβ-Cre;Ai14 mouse brain with 1 × 1 × 5 μm, as done with vascular imaging (Figure 1B). Then, we cropped out 40 ROIs with 500 × 500 × 50 (x,y,z) μm^3^ in size randomly from different areas including the cortex, hippocampus, midbrain, hypothalamus, and cerebellum. We then manually counted pericytes in 2D (5^th^ z slice from the stack) and 3D (total 10 z slices from the stack). We counted total 840 cells from 2D counting and 1769 cells from 3D counting (3D/2D ratio = 2.13 ± 0.28, mean ± standard deviation), resulting in 3D conversion factor of 2.1, which was applied as a conversion factor to estimate pericyte numbers in 3D.

To estimate the anatomical volume from each sample, the Allen CCF was registered to individual samples first using Elastix (Klein et al., 2010). Anatomical labels were transformed based on the registration parameters and the number of voxels associated with specific anatomical IDs were used to estimate the 3D volume of each anatomical area (Kim et al., 2017).

#### *In vivo* two-photon recording and comparison with STPT vascular measurement

##### Surgery

All surgeries were performed under isoflurane anesthesia (in oxygen, 5% for induction and 1.5–2% for maintenance). A custom-machined titanium head bolt was attached to the skull with cyanoacrylate glue (#32002, Vibra-tite). The head bolt was positioned along the midline and just posterior to the lambda cranial suture. Two self-tapping 3/32’’ #000 screws (J.I. Morris) were implanted into the skull contralateral to the measurement sites over the frontal lobe and parietal lobe. For measurements using two-photon laser scanning microscopy (2PLSM), a polished and reinforced thin-skull (PoRTS) window was made covering the right somatosensory cortex as described previously (Drew et al., 2010; Zhang et al., 2019). Following the surgery, mice were returned to their home cage for recovery for at least one week, and then started habituation on experimental apparatus. Habituation sessions were performed 2–4 times over the course of one week, with the duration increasing from 5 min to 45 min.

##### Measurements using two-photon laser scanning microscopy (2PLSM)

Mice were briefly anesthetized with isoflurane (5% in oxygen) and retro-orbitally injected with 50 μL 5% (weight/volume in saline) fluorescein-conjugated dextran (70 kDa, Sigma-Aldrich, cat.no.: 46945), and then fixed on a spherical treadmill. Imaging was done on a Sutter Movable Objective Microscope with a 20×, 1.0 NA water dipping objective (Olympus, XLUMPlanFLN). A MaiTai HP (Spectra-Physics, Santa Clara, CA) laser tuned to 800 nm was used for fluorophore excitation. All imaging with the water-immersion lens was done with room temperature distilled water between the PoRTS window and the objective. All the 2PLSM measurements were started at least 20 min after isoflurane exposure to reduce the disruption of physiological signals due to anesthetics. High-resolution image stacks of the vasculature were collected across a 500 by 500 μm field and up to a depth of 250 um from the pial surface. All the images were acquired with increasing laser power up to 100 mW at a depth of ~200 μm. Lateral sampling was 0.64 um per pixel and axial sampling was at 1 um steps between frames. Shortly (within 20 min) after the imaging, the mouse was perfused with FITC filling for STPT based *ex vivo* vasculature imaging.

#### *In vivo* and *ex vivo* comparison

In order to compare our measurements for vessel radii in STPT imaging datasets to vessel parameters measured *in vivo,* the same animals that were used for 2PLSM (See In vivo two-photon recording and comparison with STPT vascular measurement) underwent the FITC-fill perfusion and STPT imaging steps described above. However, STPT imaging was only conducted on the cortical hemisphere used for 2PLSM, with imaging spanning from prefrontal regions to visual cortex regions, in order to appropriately capture the primary somatosensory cortex limb region. Following stitching and tracing of the images, the raw imaging data was reconstructed in 3D in order to visualize the cortical surface. To find the 2PLSM imaging window, vessel landmarks used for navigation purpose in 2PLSM were again used to identify the same landmark vessels in the STPT imaging dataset (Figure S2). The region of interest was further confirmed by anatomical landmarks (proximity to bregma, surface vessels, etc.) through overlay of STPT and 2PLSM imaging window regions. Next, within a STPT imaging z stack, borders were inserted using ImageJ software to further outline the *in vivo* imaging window region in the 3D data. Then the *in vivo* imaging z stack data were used to identify branch points along the penetrating vessel tracked during 2PLSM. This provided identifiable characteristics to further locate the same vessel in the STPT imaging dataset. Once the exact vessel was identified in the STPT images, the precise 3D coordinates were tracked to accurately obtain the radii measurements from the traced vessel data, see the Computational: Vessel digitization/tracing section for details. In 2PLSM images, vessel diameter measurements were manually taken with adjusted pixel/micron distances using the straightline function in ImageJ. These vessel diameter measurements accounted for the lumen of the vessel, at half of the maximum fluorescence intensity profile and were adjusted for pixilation of 2PLSM data. These measurements have been further refined through VasoMetrics ImageJ macro (McDowell et al., 2021). To identify the radii and diameter measurements from the STPT imaging data, exact vessel coordinates were used to retrieve the associated vessel radii measurements using custom MATLAB code. A minimum of 10 vessel diameter measurements were taken per imaging window (each animal contained 2 imaging regions of interest) per animal.

### QUANTIFICATION AND STATISTICAL ANALYSIS

All statistical analysis, including multi-region of interest (ROI) correlation analysis, was done in Matlab (Mathworks). We used an averaged value of the experimented animals while treating each ROI as an individual data point to calculate the correlation coefficient *R* between vascular and cell density measurements. The p value was calculated based on the null hypothesis that the two groups have no correlation; the values were adjusted with the Bonferroni correction for multiple comparison correction.

## Supplementary Material

1

Video S2

Video S3

Video S4

Video S5

Table S1

Table S3

Table S4

Table S5

Table S6

Table S7

Table S9

Video S1

## Figures and Tables

**Table:** KEY RESOURCES TABLE

REAGENT or RESOURCE	SOURCE	IDENTIFIER
Chemicals, peptides, and recombinant proteins		
Tamoxifen	Sigma-Aldrich	T5648-1G; CAS: 10540-29-1
Fluorescein isothiocyanate (FITC) conjugated albumin	Sigma-Aldrich	A9771-1G; MDL: MFCD00282182
Porcine skin gelatin	Sigma-Aldrich	G1890-500G; CAS: 9000-70-8
Deposited data		
STPT full resolution images for cerebrovasculature and related cell types	This paper	https://download.brainimagelibrary.org/82/0a/820aec4a2b25b348/
Web visualization of STPT images	This paper	https://kimlab.io/brain-map/nvu/
Simulation-ready dataset for cerebrovascular tracing	This paper	Mendeley Data: https://doi.org/10.17632/mjtyry6v85.1
Cell counting data registered onto a standard reference atlas	This paper	Mendeley Data: https://doi.org/10.17632/stxvn5sv44.1
Experimental models: Organisms/strains		
C57bl/6J mice	Jackson Laboratory	Strain #: 000664;RRID:IMSR_JAX:000664
PDGFRβ-Cre mice	Volkhard Lindner Lab	N/A
Ai14 (B6.Cg-*Gt(ROSA) 26Sor^tm14(CAG-tdTomato)Hze^*/J)	Jackson Laboratory	Strain #: 007914; RRID:IMSR_JAX:007914
vGlut1-Cre (B6;129S-*Slc17a7*^*tm1.1(cre)Hze*^/J)	Jackson Laboratory	Strain #: 023527; RRID:IMSR_JAX:023527
Gad2-Cre *(Gad2*^*tm2(cre)Zjh*^/J)	Jackson Laboratory	Strain #: 010802; RRID:IMSR_JAX:010802
Ai75 (B6.Cg-Gt(ROSA) 26Sor^tm75.1(CAG-tdTomato*)Hze^/J)	Jackson Laboratory	Strain #: 025106; RRID:IMSR_JAX: 025106
nNOS-CreER (B6;129S-*Nos1^tm1.1(cre/ERT2)Zjh^*/J)	Jackson Laboratory	Strain #: 014541; RRID:IMSR_JAX:014541
Ai65 (B6;129S-*Gt(ROSA) 26Sor^tm65.1(CAG-tdTomato)Hze^*/J)	Jackson Laboratory	Strain #: 021875; RRID:IMSR_JAX:021875
PV-Flp (B6.Cg-*Pvalb*^*tm4.1(flpo)Hze*^/J)	Jackson Laboratory	Strain #: 022730; RRID:IMSR_JAX:022730
SST-Flp (*Sst*^*tm3.1(flpo)Zjh*^/J)	Jackson Laboratory	Strain #: 028579; RRID:IMSR_JAX:028579
NPY-Flp (B6.Cg-*Npy*^*tm1.1(flpo)Hze*^/J)	Jackson Laboratory	Strain #: 030211; RRID:IMSR_JAX: 030211
VIP-Flp (*Vip*^*tm2.1(flpo)Zjh*^/J)	Jackson Laboratory	Strain #: 028578; RRID:IMSR_JAX: 028578
Software and algorithms		
ImageJ	Schneider et al. (2012)	https://imagej.nih.gov/ij/; RRID:SCR_003070
VasoMetrics ImageJ macro	McDowell et al. (2021)	https://pubmed.ncbi.nlm.nih.gov/33654670/
Elastix	Klein et al. (2010)	https://elastix.lumc.nl/; RRID:SCR_009619
MatLab	Mathworks	https://www.mathworks.com/products/matlab.html; RRID:SCR_001622
Vascular tracing algorithm based on STPT imaging	This paper	ZenodoData: https://doi.org/10.5281/zenodo.6517732
Cortical Flatmap	This paper	ZenodoData: https://doi.org/10.5281/zenodo.6517175
Machine learning based cell counting algorithm	This paper	ZenodoData: https://doi.org/10.5281/zenodo.6517759
STPT imaging reconstruction algorithm	This paper	ZenodoData: https://doi.org/10.5281/zenodo.6517742
Fluid conductance simulation algorithm	This paper	ZenodoData: https://doi.org/10.5281/zenodo.6517724

## Highlights

- Resource to examine cerebrovasculature, pericytes, and related neuronal subtypes
- Dense cerebrovasculature network in motor sensory circuits
- Cortical vasculature significantly correlated with parvalbumin and nNOS neurons
- Neurovascular architecture to meet regionally distinct brain energy regulation
