# Supplementary material for: Quantitative relationship between cerebrovascular network and neuronal cell types in mice
Source: Cell Rep. Author manuscript; Available in PMC 2022 Jul 10. (PMC9271215; doi:10.1016/j.celrep.2022.110978)
Supplement: 1 [file NIHMS1818169-supplement-1.pdf]

Cell Reports, Volume *39*

Supplemental information

Quantitative relationship between cerebrovascular network and neuronal cell types in mice

Yuan-ting Wu, Hannah C. Bennett, Uree Chon, Daniel J. Vanselow, Qingguang Zhang, Rodrigo Muñoz-Castañeda, Keith C. Cheng, Pavel Osten, Patrick J. Drew, and Yongsoo Kim

Figure S1. Stitching correction, data processing pipeline, and quality control for vascular imaging, Related to Figure 1

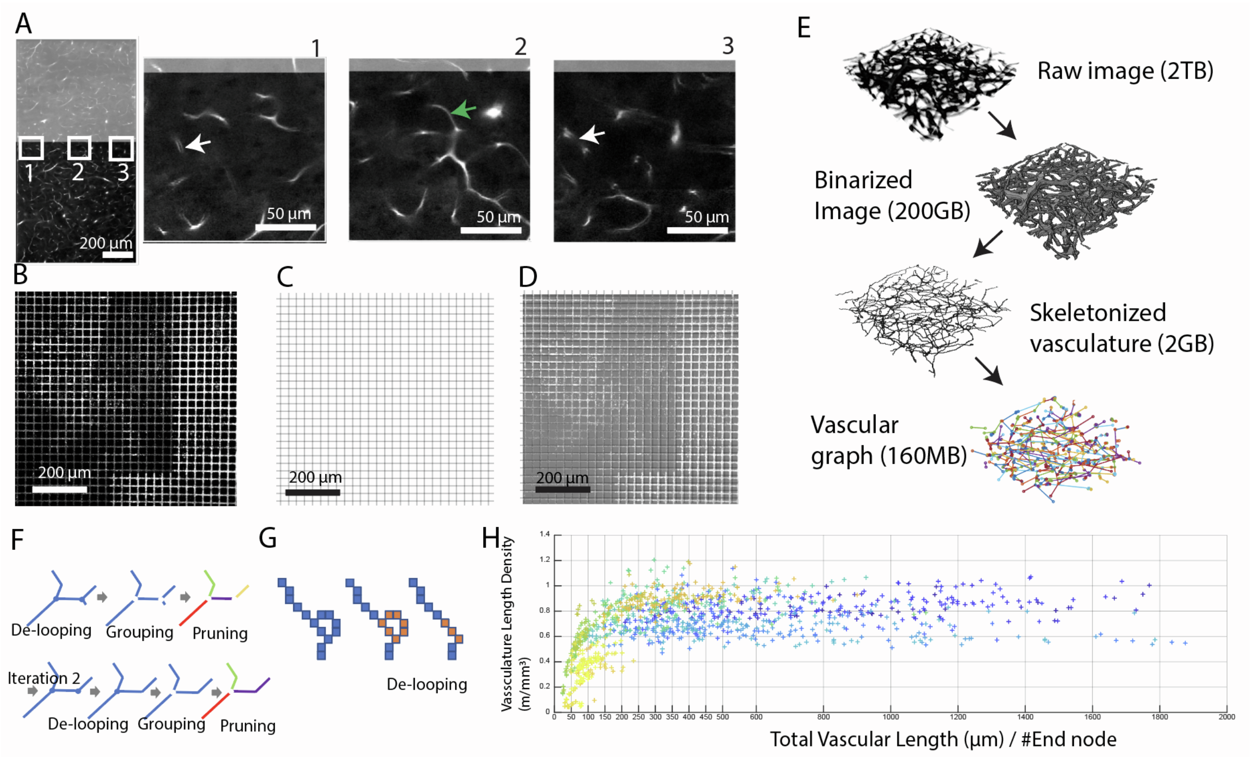

A. Correcting for optical aberration from an objective lens that impacts the vasculature tracing in two adjacent image tiles. When we manually aligned two adjacent tiles to match the middle part of the tile (e.g., a green arrow in area 2), vascular signals in the left (area 1) and right (area 3) side of tiles did not align (white arrows). **B**. The EM-grid imaged under the STPT system. The EM grid is a known orthogonal grid line, serving as a ground truth. Thus, any distortion from STPT imaged EM grid can help to assess optical aberrations introduced by the scope. **C**. A theoretical perfect gridline. **D**. Overlap of (**B**) and (**C**). Matching grid points between (**B**) and (**C**) were marked and used to calculate the deformation profile for aberration correction in our stitching algorithm. **E**. Data size throughout the analysis pipeline. **F-G**. Schematic shows how the algorithm groups, truncates the short branching, and straightens the looping artifact. The straightening showing in (**G**) is through shortest path. Those artifacts nodes that cannot be resolved this way were replaced with center representative pixel-equivalents for the later computation. The final network was processed with six such iterations. **H**. Each data point represents a ROI in one mouse. The plot includes all the animal data to check tracing quality control. The x axis is total vascular length divided by the number of end node. ROIs in the lower value in the x axis have higher probability to include higher number of dysconnectivity due to imaging artifact. The color of each data point is according with Allen ontology. We picked 250 as the threshold since the dysconnectivity no longer affected the length density after passing 250. All data points that did not pass this threshold were excluded in our final analysis.

Figure S2. *In vivo* and *ex vivo* comparison, Related to STAR Methods

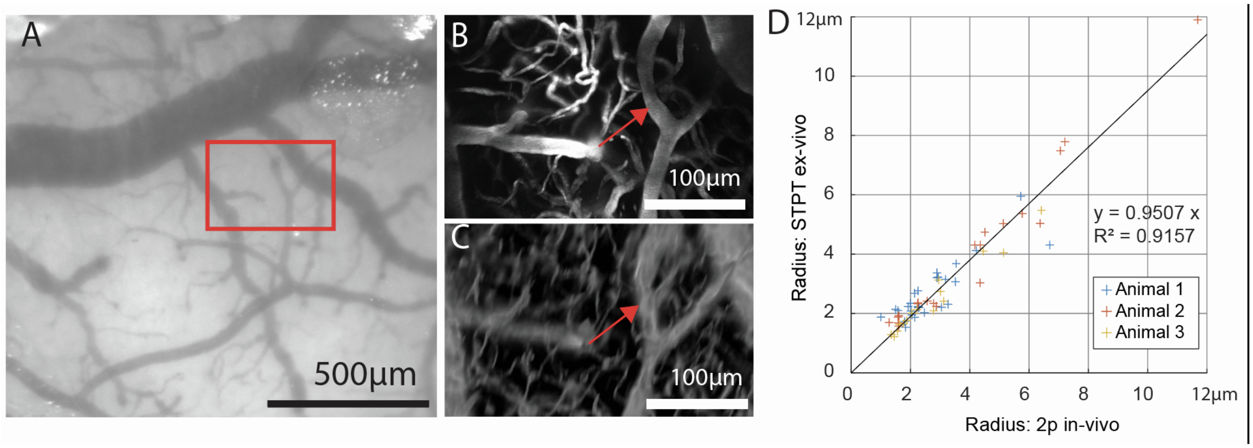

A. A zoom out view of the brain pial vessels for two-photon imaging of the areas of interest (**B**). **B**. Maximum intensity projection (200 µm thick) of *in vivo* two-photon imaging from the red boxed area. **C**. The corresponding region in the ex-vivo STPT image volume (rotated in 3D to match the imaging angle of the *in vivo* imaging). Red arrows in (**B** and **C**) indicates the same Y shaped branch identified in both imaging modalities to demonstrate preserved vascular architecture in the STPT imaging. **D**. Radius measurements from same vessels identified in both *in vivo* and *ex vivo* imaging (n=3 animals) showed near identical results.

Figure S3. Deep Learning Neural Network for pericyte and nNOS cell counting, Related to STAR Methods

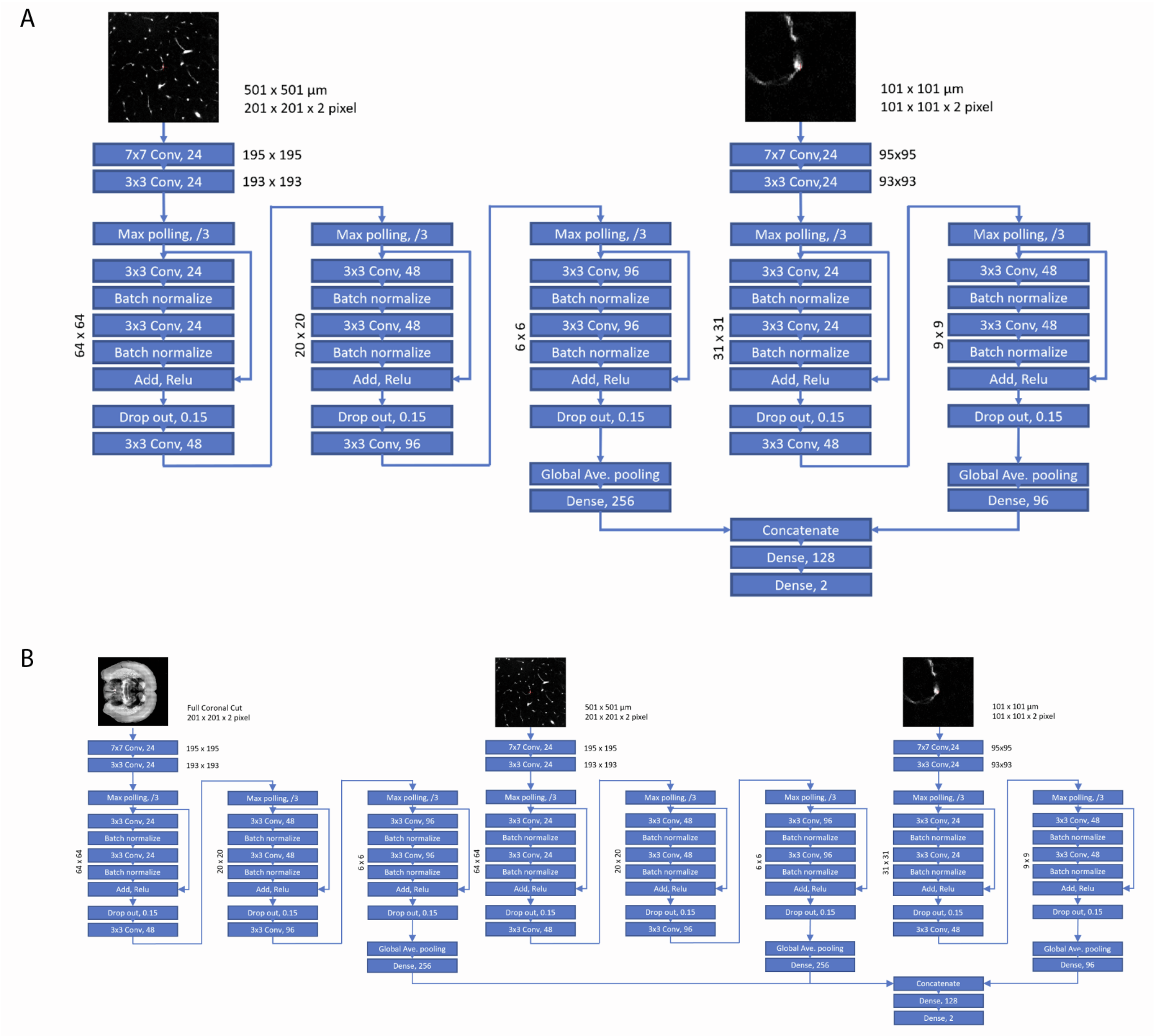

A. The network used in pericyte counting. **B**. The network used in nNOS and its subtype counting. Both networks are adapted version of RESNET with concatenating of multiple resolution images to bring the awareness of the nature of the cell to the network; Such as its shape in high resolution, the neighboring background feature in mid resolution, and the brain location in the low resolution.

Figure S4. Low fluid conductance and pericyte densities in the hippocampal areas, Related to Figure 3 and 4.

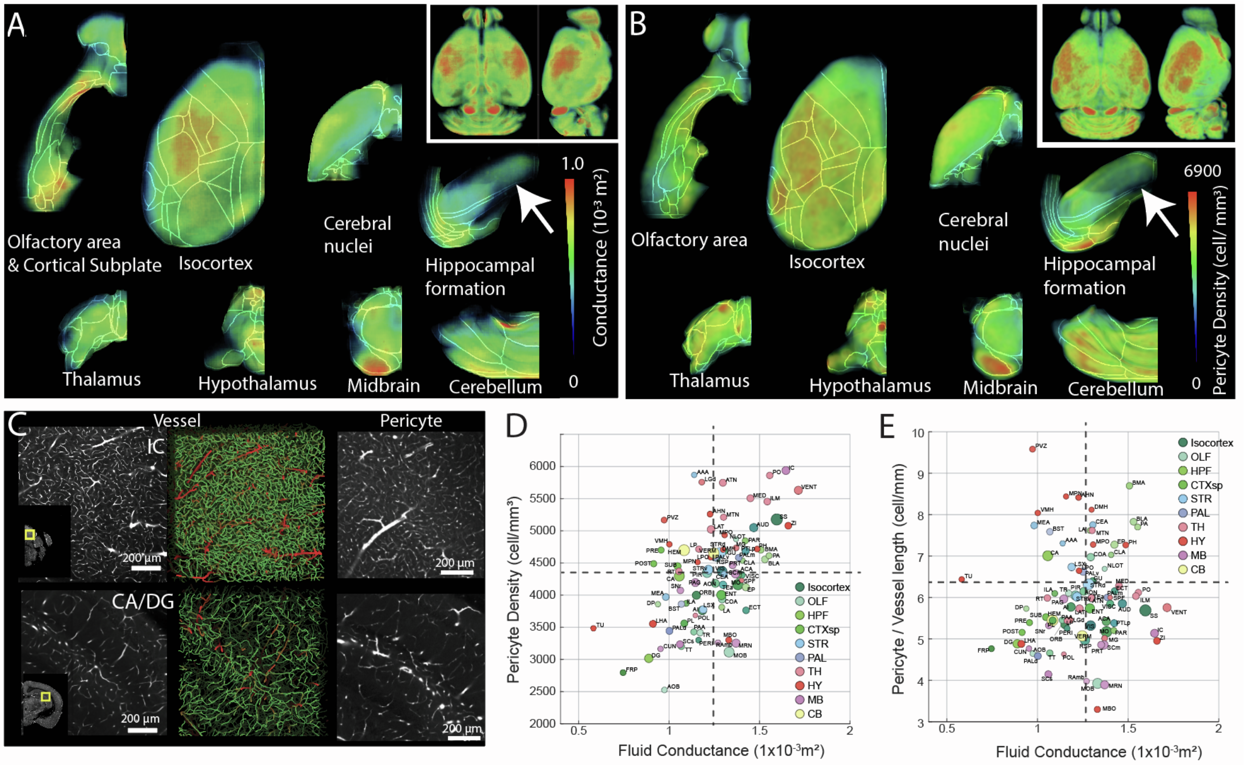

A-B. Graphical representation of spherically integrated fluid conductance results (**A**) and pericyte density (**B**) of the whole brain. White arrows highlight relatively low fluid conductance and pericyte densities in the hippocampal areas. See also Table S4 and S7. **C**. Examples of high densities of vasculature and pericytes in the inferior colliculus (IC) and Ammon's horn/dentate gyrus (CA/DG) of the hippocampus. Mid panels represent detected vasculature in 100 µm thick sections (green: microvessels, red: large vessels). **D-E**. Scatter plot of pericyte and fluid conductance (**D**), and pericyte vascular coverage (pericyte/vessel length) and fluid conductance (**E**).

Table S2. Cell type specific transgenic mouse lines to label mural and neuronal cell types. Related to Figure 1

Cell Type	Transgenic mouse lines
Mural cell types including Pericyte	PDGFRb-Cre;Ai14
pan nNOS neurons	nNOS-CreER;Ai14
nNOS subtype co-expressing NPY	nNOS-CreER;NPY-Flp:Ai65
nNOS subtype co-expressing SST	nNOS-CreER;SST-Flp:Ai65
nNOS subtype co-expressing PV	nNOS-CreER;PV-Flp:Ai65
nNOS subtype co-expressing VIP	nNOS-CreER;VIP-Flp:Ai65
pan glutamatergic neurons	vGlut1-Cre;Ai75
pan GABAergic neurons	Gad2-Cre;Ai75
GABAergic subtype expressing PV	PV-Cre;H2B-GFP
GABAergic subtype expressing SST	SST-Cre;H2B-GFP
GABAergic subtype expressing VIP	VIP-Cre;H2B-GFP

Table S8. Density of the cerebrovasculature, pericytes, and neuronal subtypes in the isocortex. Related to Figure 5

Region of Interest	Vessel length density (m/mm^3)	Pericyte (cell/mm^3)	nNOS neurons (cell/mm^3)	PV neurons (cell/mm^3)	vGlut neurons (cell/mm^3)
Motor	0.81 ± 0.05	4,164 ± 308	104 ± 17	2,678 ± 203	35,566 ± 823
Somatosensory	0.91 ± 0.07	5,177 ± 466	108 ± 16	3,664 ± 281	45,022 ± 838
Auditory	0.86 ± 0.06	5,049 ± 535	121 ± 16	2,965 ± 295	42,178 ± 1,676
Visual	0.82 ± 0.07	4,071 ± 440	112 ± 22	2,927 ± 352	49,329 ± 1,132
Retrosplenial	0.92 ± 0.07	4,598 ± 548	103 ± 20	3,330 ± 387	44,631 ± 2,494
Posterior Parietal	0.87 ± 0.07	4,684 ± 404	91 ± 17	3,220 ± 255	47,030 ± 875
Orbital	0.75 ± 0.08	4,685 ± 404	106 ± 17	2,290 ± 320	38,014 ± 1,559
Anterior Cingulate	0.8 ± 0.05	4,324 ± 350	154 ± 32	2,371 ± 213	41,208 ± 1,039
Prelimbic	0.65 ± 0.04	3,560 ± 458	171 ± 34	1,199 ± 171	39,270 ± 1,098
Infralimbic	0.64 ± 0.02	3,872 ± 380	232 ± 38	503 ± 120	43,679 ± 1,973
Visceral	0.74 ± 0.04	4,312 ± 434	177 ± 33	1,846 ± 204	37,081 ± 1,546
Gustatory	0.73 ± 0.03	4,681 ± 366	194 ± 34	1,652 ± 212	40,783 ± 1,467
Agranular Insular	0.65 ± 0.05	3,763 ± 315	207 ± 41	1,154 ± 118	31,290 ± 1,023
Perirhinal	0.63 ± 0.03	3,300 ± 388	265 ± 69	979 ± 142	24,789 ± 3,196
Temporal Association	0.7 ± 0.04	4,160 ± 403	187 ± 37	1,189 ± 210	42,407 ± 1,907
Ectorhinal	0.61 ± 0.02	3,763 ± 321	283 ± 63	623 ± 117	38,578 ± 3,400

Number = average ± standard deviation, see Table S1, S4, S5, S6 for full dataset.
